# The exosome-mediated autocrine and paracrine actions of plasma gelsolin in ovarian cancer chemoresistance

**DOI:** 10.1038/s41388-019-1087-9

**Published:** 2019-11-07

**Authors:** Meshach Asare-Werehene, Kiran Nakka, Arkadiy Reunov, Chen-Tzu Chiu, Wei-Ting Lee, Mohammad R. Abedini, Pei-Wen Wang, Dar-Bin Shieh, F. Jeffrey Dilworth, Euridice Carmona, Tien Le, Anne-Marie Mes-Masson, Dylan Burger, Benjamin K. Tsang

**Affiliations:** 1 Departments of Obstetrics & Gynecology and Cellular & Molecular Medicine, University of Ottawa, Ottawa, ON Canada; 2 Chronic Disease Program and Regenerative Medicine Program, Ottawa Hospital Research Institute, Ottawa, ON K1H 8L6 Canada; 3 Departments of Medicine and Cellular & Molecular Medicine, University of Ottawa, Ottawa, ON Canada; 4 Department of Biology, St. Francis Xavier University, 2320 Notre Dame Avenue, Antigonish, NS B2G 2W5 Canada; 5 Institute of Basic Medical Science, Institute of Oral Medicine and Department of Stomatology, National Cheng Kung University Hospital, National Cheng Kung University, Tainan, 704 Taiwan; 6 Cellular and Molecular Medicine Research Center, Department of Pharmacology, Birjand University of Medical Sciences, Birjand, 97178 Iran; 7 Advanced Optoelectronic Technology Center and Center for Micro/Nano Science and Technology, National Cheng Kung University, Tainan, 701 Taiwan; 8 Centre de recherche du CHUM et Institut du cancer de Montréal, Montréal, QC H2X 0A9 Canada; 9 Department of Medicine, Université de Montréal, Montréal, QC H3C 3J7 Canada

**Keywords:** Ovarian cancer, Extracellular signalling molecules

## Abstract

Ovarian cancer (OVCA) is the most lethal gynecological cancer, due predominantly to late presentation, high recurrence rate and common chemoresistance development. The expression of the actin-associated protein cytosolic gelsolin (GSN) regulates the gynecological cancer cell fate resulting in dysregulation in chemosensitivity. In this study, we report that elevated expression of plasma gelsolin (pGSN), a secreted isoform of GSN and expressed from the same GSN gene, correlates with poorer overall survival and relapse-free survival in patients with OVCA. In addition, it is highly expressed and secreted in chemoresistant OVCA cells than its chemosensitive counterparts. pGSN, secreted and transported via exosomes (Ex-pGSN), upregulates HIF1α–mediated pGSN expression in chemoresistant OVCA cells in an autocrine manner as well as confers cisplatin resistance in otherwise chemosensitive OVCA cells. These findings support our hypothesis that exosomal pGSN promotes OVCA cell survival through both autocrine and paracrine mechanisms that transform chemosensitive cells to resistant counterparts. Specifically, pGSN transported via exosomes is a determinant of chemoresistance in OVCA.

## Introduction

Chemoresistance is a major obstacle in the treatment of ovarian cancer (OVCA), one of the most fatal gynecological cancers. Although most patients initially respond to platinum-based chemotherapy, about 70–80% of the tumor relapses and become resistant to treatment especially with the high-grade serous (HGS) histological subtype　[1]. The HGS subtype is considered as the most fatal with the worst mortality compared with the other subtypes [2]. Platinum or taxane derivatives combined with cytoreduction are standard first-line treatment strategy for OVCA; however, there has been no significant change in a 5-year patient survival rate, due to high rate of tumor recurrence and chemoresistance [2]. It is therefore urgent to investigate novel targets and markers that are involved in OVCA recurrence and chemoresistance and how these affect patient survival. The molecular and cellular mechanisms underpinning chemoresistance are multifactorial, as they involve apoptosis evasion, aberrant expression and activation of survival factors, dysregulation of tumor suppressors and impairment of the immunological defence system [3–5].

Gelsolin (GSN), an 80–85 kDa calcium-dependent multifunctional actin-binding protein, has two well-characterized isoforms—cytoplasmic gelsolin (cGSN) and plasma gelsolin (pGSN) [6]. There are other isoforms that are less characterized. These isoforms are encoded by a single gene on chromosome 12 as a result of alternative splicing and different transcriptional initiation sites [6]. The extra 25 amino acid sequence at the N-terminal of pGSN is one of the main architectural differences that distinguish it from cGSN [7]. GSN forms a complex with FLICE-like inhibitory protein (FLIP) and Itch and stabilizes FLIP in a nonstress state, whereas cisplatin (CDDP) dissociates GSN from the complex in chemosensitive cells, thereby facilitating FLIP ubiquitination and degradation, caspase-3 activation, and GSN cleavage [8]. We have recently shown that cGSN overexpression correlates with chemoresistance, poor prognosis, aggressive behavior, and cancer death [9], whereas the role of pGSN remain elusive.

pGSN is an extracellular actin scavenger with an average concentration at ~200–300 µg/ml, which is mostly produced by the muscle and distributed by body fluids [6, 10–13]. pGSN has been implicated in various inflammatory disorders, injuries, and bacterial infections [6, 14] although its mechanistic involvement is poorly understood. High levels of pGSN have also been detected in the plasma/serum and tissues of head-and-neck, colorectal, prostate and, breast cancers [13]; however, the exact mechanism is largely unknown. Although pGSN has been shown to interact with α5β1 integrin [15], the detailed interaction and its consequence on chemosensitivity in OVCA are yet to be studied. To date, there is no data on the possible role of pGSN in OVCA recurrence, suboptimal surgical debulking, and chemoresistance.

Extracellular vesicles (EVs) play a key role in cell–cell communication through surface interactions and the transfer of proteins, nucleic acids, and fatty acids [16–19]. Exosomes (EXs) are vesicles of ~30–100 nm in size and formed within endosomes by membrane invaginations, whereas microvesicles (MVs) range from 0.1 to 1.0 µm and are produced by membrane blebbing in cells under stress [17, 18]. The secretion of EXs or MVs from cancer cells could regulate the functions of neighboring cells, including noncancerous or immune cells [17, 19–21]. EVs are believed to play a role in cancer progression, survival, and metastasis although their participation in cellular basis of chemoresistance in OVCA remains largely unknown [17]. Whether EXs containing pGSN (Ex-pGSN) may be important in the regulation of chemosensitivity in neighboring OVCA cells has not been reported. In this study, we report for the first time pGSN secretion and transport via EXs, its functional interaction with HIF1α in an autocrine manner and conferring of cisplatin resistance in otherwise chemosensitive OVCA cells.

## Results

### pGSN expression in ovarian cancer patients predicts clinical outcomes

**Fig. 1:**
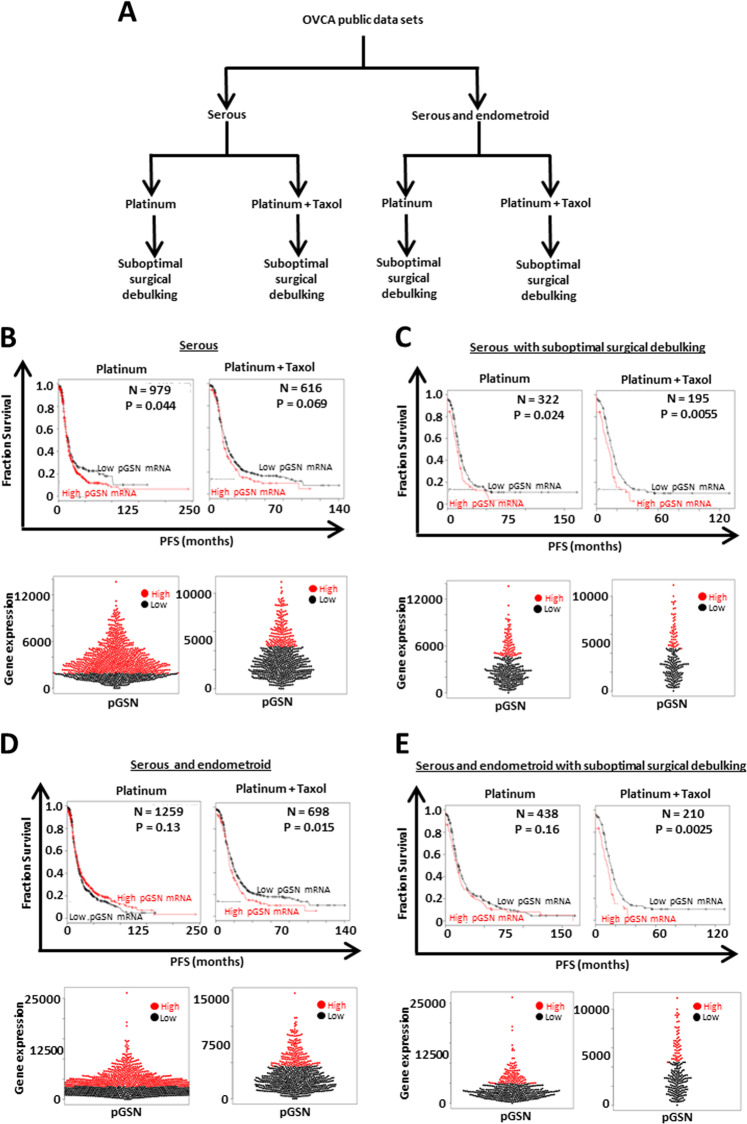
High pGSN expression is associated with tumor recurrence in patients with ovarian cancer. **a** Ovarian cancer public datasets were stratified using histological subtype (serous and endometroid), chemotherapeutic agents, and suboptimal surgical debulking. Kaplan–Meier survival analysis and beeswarm plots with optimal cutoff values of pGSN expression were performed on **b** only serous patients, **c** serous and endometroid patients, **d** serous patients with suboptimal surgical debulking, and **e** serous and endometroid patients with suboptimal surgical debulking with treatments containing either platinum or platinum + taxol. *P* values were calculated by the log-rank test

Using meta-analysis, we assessed the expression of pGSN with the 200696_s_at probe (Supplementary Tables 1 and 2) in primary ovarian tumor (serous and endometroid) in the context of the patient’s clinical outcomes by interrogating publicly available gene expression datasets using Kaplan–Meier plotter OVCA survival analysis (www.kmplot.com) [22]. pGSN gene expression was stratified by histological subtype (serous; serous and endometroid), chemotherapeutic agents (platinum or platinum + taxol), and suboptimal surgical debulking (Fig. 1a) and correlated with progression-free survival (PFS) [22]. In serous patients with treatments containing platinum derivatives, elevated expression of pGSN was significantly (*p* = 0.044) associated with shorter time for tumor recurrence (16.6 months) compared with patients with lower pGSN expression (18.27 months) (Fig. 1b). The tumor recurrence trend was similar in serous patients with treatments containing both platinum and taxol agents although the difference was not significant (*p* = 0.069; low pGSN, 17.38 months; high pGSN, 14.9) (Fig. 1b). Analysis of serous patients with suboptimal surgical debulking revealed that increased pGSN expression was significantly correlated with shorter time for tumor recurrence irrespective of treatment component [(platinum, *p* = 0.024; low pGSN, 15.01 months; high pGSN, 13 months), (platinum and taxol, *p* = 0.0055; low pGSN, 15.01 months; high pGSN, 11.93 months)] (Fig. 1c).

Our interrogation of both serous and endometroid datasets revealed that patients treated with platinum and taxol compounds and had elevated expression of pGSN experienced significantly shortened PFS (*p* = 0.015; low pGSN, 18 months; high pGSN, 14.87 months) (Fig. 1d). However, no significant difference was observed in the same datasets with treatments containing only platinum derivatives (*p* = 0.13; low pGSN, 19 months; high pGSN, 19.3 months) (Fig. 1d). When the datasets (serous and endometroid) were stratified using suboptimal surgical debulking and treatment containing platinum and taxol, there was significantly shorter time to occurrence in patients with elevated levels of pGSN (*p* = 0.0025; low pGSN, 15.01 months; high pGSN, 11.93 months) (Fig. 1e). In the context of patients treated with platinum derivatives, we observed that elevated pGSN expression was associated with shorter PFS (14.9 months) compared with those with lower pGSN expression (PFS; 16.83 months) although the difference was not significant (*p* = 0.16) (Fig. 1e). The beeswarm plot further provided a visual view of the relative expression of pGSN in OVCA patients dichotomized as either high or low (Fig. 1b–e; bottom panels). Although not shown by any figure, there were no significant differences between overall survival (OS) and pGSN levels irrespective of stratification. We therefore decided not to present the OS data in the current study.

### pGSN content and secretion are higher in chemoresistant OVCA cells and are associated with decreased CDDP-induced apoptosis

**Fig. 2:**
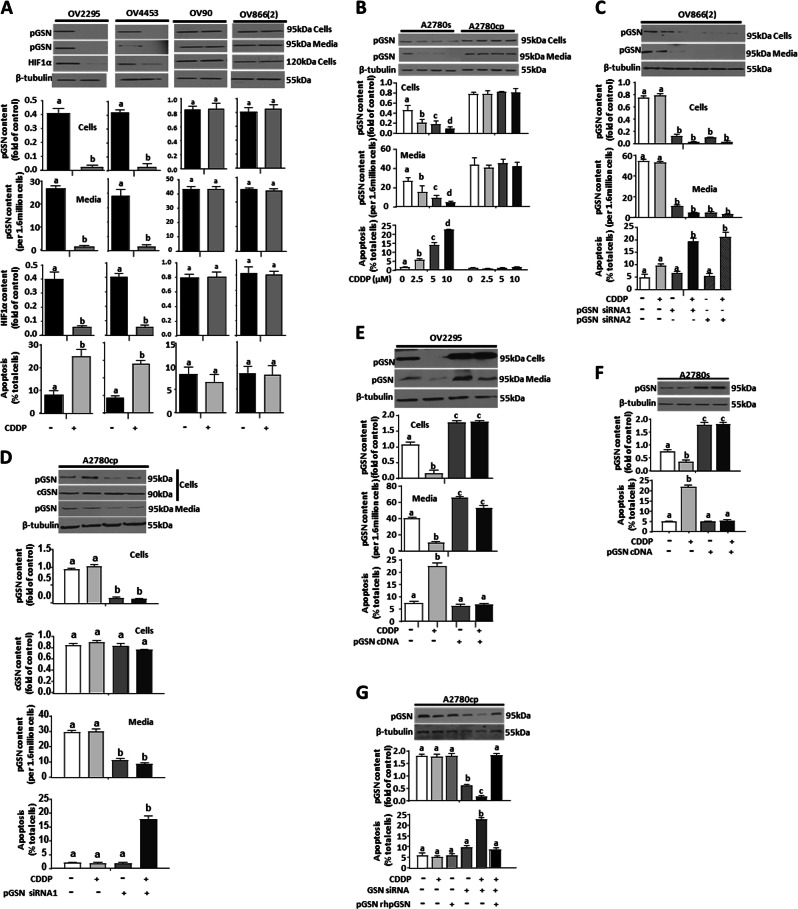
pGSN regulates CDDP-induced apoptosis in OVCA cells. **a**, **b** CDDP decreased pGSN content and induced apoptosis in chemosensitive (OV2295, OV443, and A2780s) but not chemoresistant (OV90, OV866(2), and A2780cp) OVCA cells. OVCA cells were cultured with or without CDDP (10 µM; 24 h). **c**, **d** Silencing pGSN in OV866(2) and A2780cp cells sensitized them to CDDP-induced apoptosis. OV8669(2) and A2780cp cells were transfected with pGSN siRNA (50 nM, 24 h; which specifically knocked down pGSN but not cGSN), and then treated with or without CDDP (10 µM; 24 h). **e**, **f** Overexpression of pGSN cDNA attenuated CDDP-induced apoptosis in OV2295 and A2780s cells. OV2295 and A2780s cells were transfected with pGSN cDNA (2 µg; 24 h) and cultured with or without CDDP (10 µM; 24 h). **g** A2780cp cells (with total GSN knocked down) were cultured with rhpGSN (10 µM; 24 h) before treatment with CDDP (0 and 10 µM; 24 h). pGSN, cGSN, and β-tubulin (loading control) contents were assessed by western blotting (WB) and apoptosis determined morphologically by Hoechst 33258 DNA staining. [**a** (a; ***p < 0.001 vs b, c, and d); **b** (a; ***p < 0.001 vs b); **c** (a; ***p < 0.001 vs b and c); **d** (a; ***p < 0.001 vs b); **e** (a; ***p < 0.001 vs b and c); **f** (a; ***p < 0.001 vs b and c); **g** (a; ***p < 0.001 vs b and c);]. *N* = 3

To examine the mechanistic action of pGSN in the regulation of chemosensitivity in OVCA cells, we compared the influence of *Cis*-diaminedichloroplatinum (CDDP) on pGSN levels in chemosensitive and resistant OVCA cells of HGS subtype with various p53 mutational status and extended these investigations to include the OVCA of the endometroid subtypes (see Supplementary Table 3). HGS [chemosensitive (OV2295 and OV4453) and chemoresistant (OV90, OV866(2) and Hey] and endometroid [chemosensitive (A2780s and PA-1) and chemoresistant (A2780cp and SKOV-3)] OVCA cells were cultured with or without CDDP (10 µM; 24 h) and cellular and conditioned media contents of pGSN were assessed by WB and ELISA. Cellular and secreted pGSN in the resistant HGS cells (OV90, Hey, OV866(2)) were not affected by CDDP treatment although their contents decreased in the chemosensitive HGS cell lines (OV2295 and OV4453) (Figs. 2a and S1A). CDDP-induced apoptosis in the chemosensitive HGS cells but not the resistant phenotypes (Figs. 2a and S1A). Likewise, pGSN content in OVCA cells of endometroid subtypes was expressed and secreted in larger amounts in the chemoresistant cells than their sensitive counterparts, irrespective of their p53 status (Figs. 2b, S1B, C and S2). CDDP decreased cellular and secreted pGSN contents in the CDDP-sensitive cells but not in the resistant cells (Figs. 2a, b, S1 and S2). CDDP treatment induced concentration-dependent apoptosis in chemosensitive cells but not in the resistant cells (***p < 0.001) (Figs. 2a, b and S1) suggesting a possible association between pGSN overexpression and OVCA chemoresistance.

### pGSN is involved in the regulation of CDDP sensitivity in OVCA cells

**Fig. 3:**
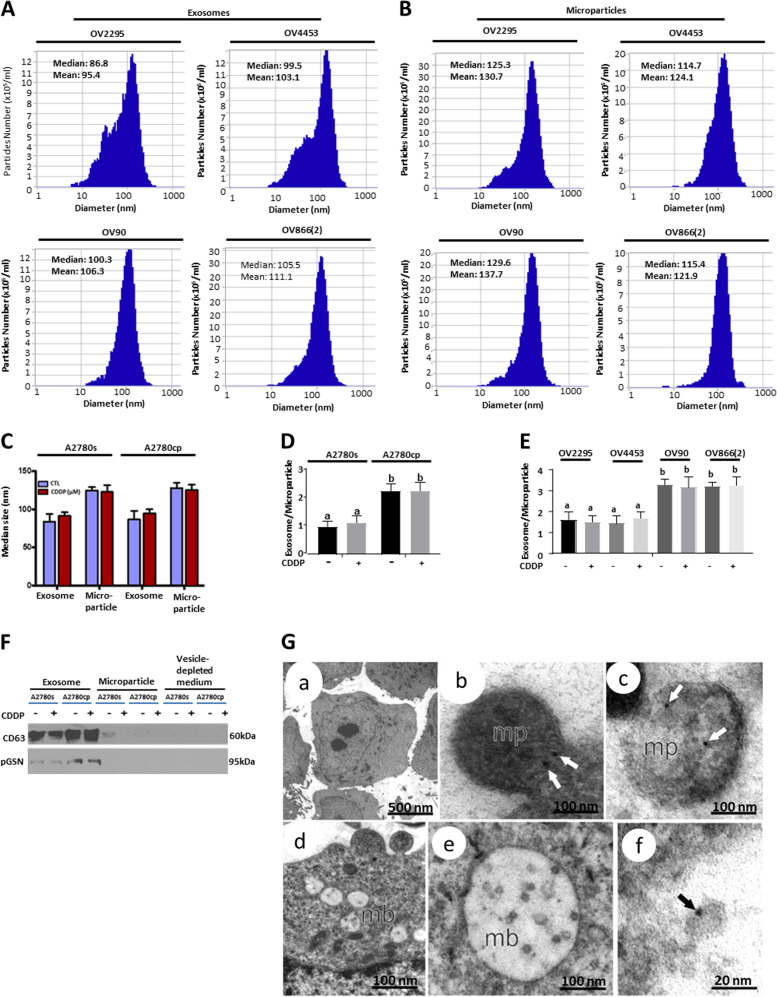
Extracellular vesicle characterization and CDDP effect on vesicle size distribution. HGS chemosensitive and resistant cells secrete exosomes (**a**) and microparticles (**b**) as confirmed by nanoparticle tracking. **c** A2780s and A2780cp cells secrete both exosomes and microparticles. OVCA cells were cultured with or without CDDP (10 µM; 24 h). Exosomes and microparticles were isolated from their conditioned media by ultracentrifugation and characterized by Nanoparticle Tracking Analyser. **d**, **e** The exosome-to-microparticle ratio is higher in chemoresistant cells compared with chemosensitive cells; their concentrations were not affected by CDDP treatment. **f** pGSN is predominantly identified in the exosomes compared with microparticles of A2780s and A2780cp; however, pGSN content is higher in the A2780cp cells compared with A2780s cells. Exosomes and microparticles were isolated as described above and pGSN and CD63 (exosome marker) contents assessed by WB. **g** Electron micrograph showing pGSN in microparticles (mp) and multivesicular bodies/exosomes (mb); white and black arrows showing pGSN in mp and mb, respectively. pGSN in fixed A2780cp cells was immunostained with 18-nm colloidal gold particles, observed and photographed with a Jeol JEM 1230 transmission electron microscope. Scale bars, 500 nm (**a**), 100 nm (**b–e**) and 20 nm (**f**). [**d** (a; **p < 0.01 vs b); **e** (a; **p < 0.01 vs b)]. *N* = 3

To further examine whether CDDP responsiveness of OVCA cells is regulated by pGSN, chemoresistant OVCA cells [OV866(2) (Fig. 2c), Hey (Fig. S3A), and A2780cp (Figs. 2d and S3B) were transfected with either pGSN or scramble (control) small interfering RNAs (siRNA; 50 nM; 24 h) and treated with CDDP (0 and 10 µM; 24 h) to determine if pGSN knockdown would sensitize the chemoresistant OVCA cells to CDDP-induced apoptosis. pGSN knockdown resulted in the sensitization of the resistant cells to CDDP-induced apoptosis (10 µM; Figs. 2c, d and S3B; ***p < 0.001). Moreover, chemosensitive OV2295 and A2780s cells transfected with pGSN cDNA (empty vector as control; 1 µg; 24 h) and subsequently treated with CDDP (0 and 10 µM; 24 h) exhibited significant attenuation in CDDP-induced apoptotic response (***p < 0.001; Fig. 2e, f). A2780cp cells treated with GSN siRNA (known to downregulate both cGSN and pGSN) were reconstituted with recombinant human plasma gelsolin (rhpGSN), and then treated with CDDP. pGSN content in A2780cp cells was significantly decreased (both in the cells and conditioned media) by GSN siRNA and also sensitized the resistant cells to CDDP-induced apoptosis (10 µM; ***p < 0.001; Fig. 3g). rhpGSN reconstitution (10 µM; 24 h) attenuated CDDP-induced apoptosis in A2780cp cells, in which GSN was knocked down (Fig. 3g), suggesting that the antiapoptosis response of GSN was primarily that of the pGSN. Taken together, these findings suggest that pGSN plays a key role in OVCA responsiveness to CDDP and its downregulation may present as an opportunity to sensitize chemoresistant OVCA cells to CDDP-induced apoptosis.

### pGSN is transported by exosomes which auto-upregulate pGSN content through α5β1 integrin signaling

To investigate the mode of secretion and extracellular transport of pGSN, chemosensitive (OV2295, OV4453, A2780s, and PA-1), and chemoresistant (OV90, OV866(2), A2780cp, and Hey) OVCA cells were cultured in the absence and presence of CDDP (10 µM; 24 h) and EVs (EXs and microparticles) in conditioned media were isolated and characterized by WB, NTA, and immunoelectron microscopy (iEM). Although both the sensitive and resistant cells secreted both types of EVs (Figs. 3a–c and S4A, B), the resistant cells secreted significantly more EXs compared with their sensitive counterparts (Fig. 3d, e). Regardless of the CDDP sensitivity of the cells EX secretion was not affected by CDDP treatment (Fig. 3c–e). Microparticles blebbed from the cell membrane with an average size of 500 nm, whereas EXs were formed in multivesicular bodies with an average size of 100 nm. pGSN was detected in both EXs and microparticles using 18-nm colloidal gold particles (Fig. 3g). Chemoresistant cells-derived EXs contained increased levels of pGSN compared with microparticles (Fig. 3f). Exosomal pGSN was secreted in greater amounts in the resistant cells compared with their sensitive counterparts (Fig. 3f).

**Fig. 4:**
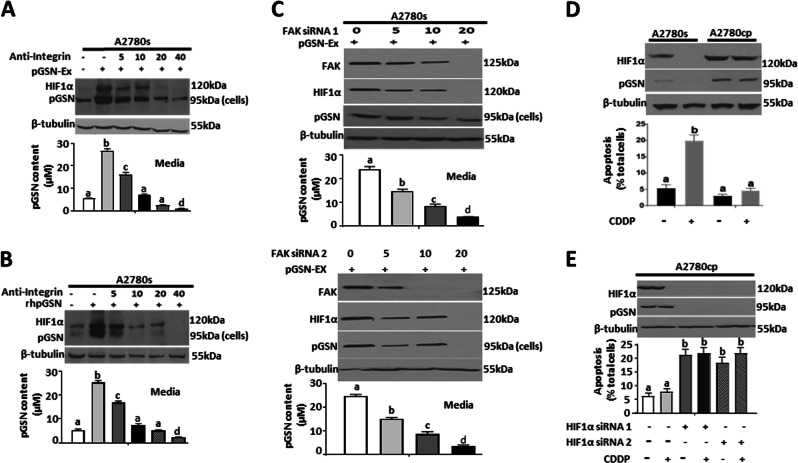
The integrin signaling pathway is involved in the autocrine upregulation of pGSN content by pGSN. **a**, **b** The α5β1 integrin receptor blocker ATN 161 attenuates the upregulation of pGSN by Ex-pGSN (**a**) and rhpGSN (**b**). A2780s cells were treated with anti-α5β1 integrin (40 µM; 3 h) followed by A2780cp-derived Ex-pGSN (40 µg/400,000 cells; 24 h) or rhpGSN (10 µM; 24 h). **c** Knockdown of FAK resulted in the downregulation of HIF1α and pGSN contents. A2780s cells were transfected with FAK siRNA1 and siRNA2 (0–20 pmol; 24 h) before culture with A2780cp-derived Ex-pGSN (40 µg/400,000 cells; 24 h). **d** pGSN and HIF1-α contents were higher in A2780cp cells compared with A2780s cells; CDDP reduced their content in A2780s but not in A2780cp cells. CDDP-induced apoptosis in A2780cp cells was higher than that in A2780s cells. A2780s and A2780cp cells were cultured with or without CDDP (10 µM; 24 h). **e** HIF1-α silencing reduced the content of pGSN and sensitized A2780cp cells to CDDP-induced apoptosis. A2780cp cells were transfected with HIF1α siRNA1 and siRNA2 (200 pmol; 24 h) before culture with or without CDDP (10 µM; 24 h). pGSN, FAK, HIF1α, and β-tubulin (loading control) contents were assessed by WB and apoptosis determined morphologically by Hoechst 33258 DNA staining. pGSN levels in the conditioned media were assessed by the sandwich ELISA. [**a** (a; ***p < 0.001 vs b, c, d); **b** (a; ***p < 0.001 vs b, c, d); **c** (a; ***p < 0.001 vs b, c, d); **d** (a; ***p < 0.001 vs b); **e** (a; ***p < 0.001 vs b)]. *N* = 3

Despite the observation that pGSN interacts with α5β1 integrin receptor [15], whether and how this interaction regulates chemosensitivity in OVCA remains to be demonstrated. To investigate this possibility, A2780s cells were treated with the α5β1 integrin inhibitor ATN 161 (0 and 40 µM; 3 h) or FAK siRNA (0 and 20 pmol; 24 h) before treatment with Ex-pGSN (40 µg; 24 h) or exogenous human rhpGSN (10 µM; 24 h) (Fig. 4a–c). Ex-pGSN and exogenous pGSN significantly increased the contents of endogenous pGSN and HIF1α; however, these responses were attenuated by the presence of the α5β1 integrin receptor antagonist and FAK downregulation (Fig. 4a–c). The downregulation of endogenous pGSN also resulted in decreased levels of secreted pGSN (Fig. 4a–c). This is suggestive that the α5β1 integrin signaling pathway is involved in the autocrine-mediated upregulation of endogenous pGSN content.

### Akt and HIF1α upregulation promotes the expression and antiapoptotic action of pGSN on CDDP-induced apoptosis

**Fig. 5:**
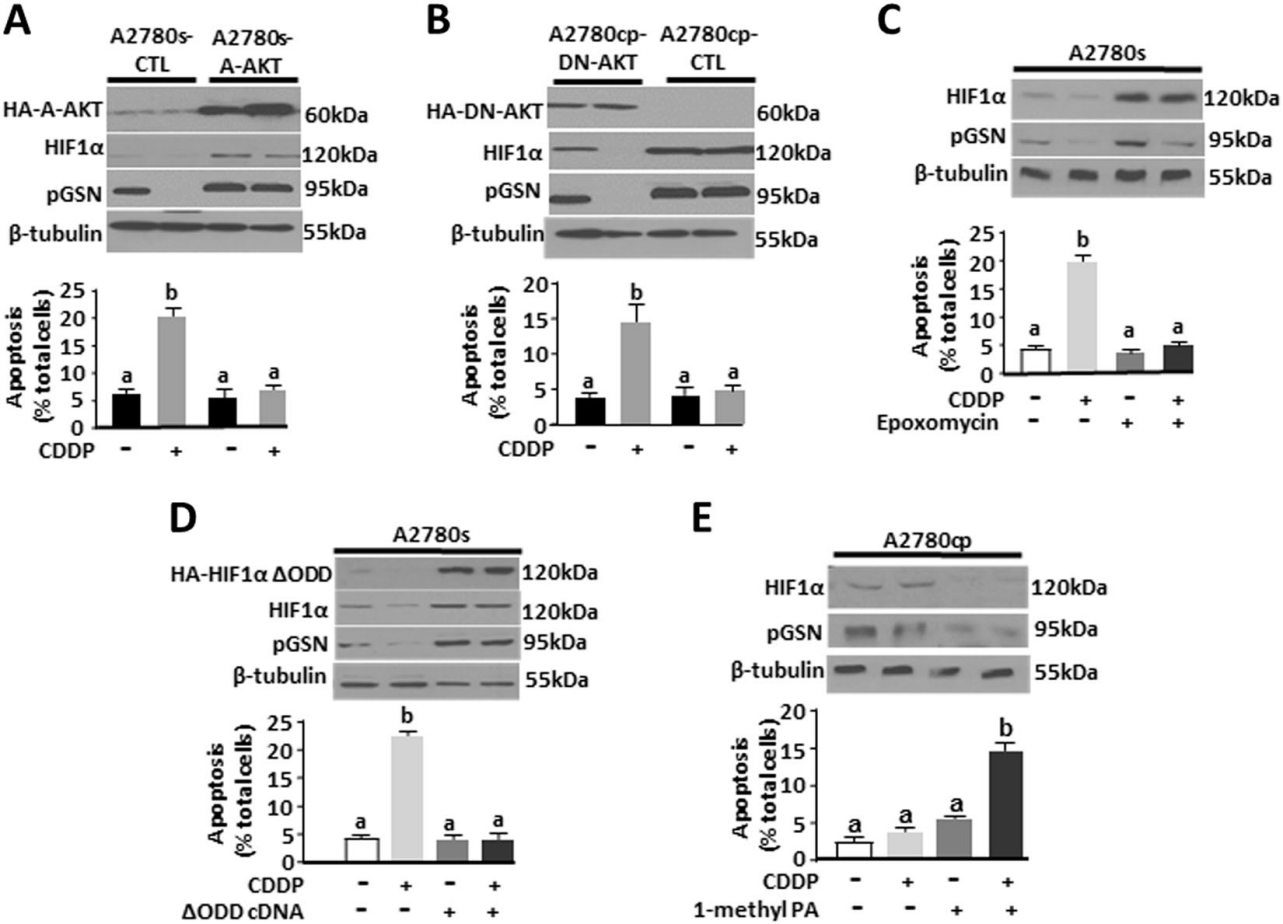
pGSN-mediated OVCA chemoresistance involves HIF1α modulation by Akt. **a** Activation of Akt in chemosensitive cells increases the contents of pGSN and HIF1α and renders them resistant to CDDP-induced apoptosis. A2780s cells constitutively expressing an activated Akt (A2780s-A-AKT) and its scrambled control cells (A2780s-CTL) were cultured with or without CDDP (10 µM; 24 h). **b** pGSN and HIF1α contents are decreased and CDDP-induced apoptosis enhanced in chemoresistant cells when Akt function is downregulated. A2780cp cells constitutively expressing triple-mutant dominant Akt (A2780cp-DN-AKT) or scrambled control (A2780cp-CTL) cultured with or without CDDP (10 µM; 24 h). **c** Inhibition of proteasomal degradation of HIF1α in chemosensitive cells increases the content of pGSN and attenuates CDDP-induced apoptosis. A2780s cells were pretreated with epoxomycin (10 nM; 3 h) and cultured with or without CDDP (10 µM; 24 h) in the presence of the inhibitor. **d** Forced expression of a proteasomal nondegradable mutant of HIF1α increases pGSN content and inhibits CDDP-induced apoptosis in chemosensitive cells. A2780s cells were transfected with ΔHIF1α cDNA (1 µg; 24 h) and treated with or without CDDP (10 µM; 24 h). **e** Induction of proteasomal HIF1α degradation in chemoresistant cells decreases pGSN content and renders them sensitive to CDDP-induced apoptosis. A2780cp cells were pretreated with the proteasome activator 1-methyl PA (10 µM; 3 h), and then cultured with or without CDDP (10 µM; 24 h) in the presence of the activator. pGSN, HIF1α, and β-tubulin (loading control) contents were assessed by WB and apoptosis determined morphologically by Hoechst 33258 DNA staining. [**a** (a; ***p < 0.001 vs b); **b** (a; ***p < 0.001 vs b); **c** (a; ***p < 0.001 vs b); **d** (a; ***p < 0.001 vs b); **e** (a; ***p < 0.001 vs b)]. *N* = 3

As observed with pGSN, HIF1α was also highly expressed in the resistant cells compared with their sensitive counterparts; CDDP decreased HIF1α content and induced apoptosis in the CDDP-sensitive but not resistant cells (Fig. 4d). HIF1α downregulation by siRNA (200 pmol; 24 h) decreased pGSN content in A2780cp cells and sensitized them to CDDP-induced apoptosis (*p* < 0.001) (Fig. 4e). We further analyzed the possibility that the expression and antiapoptotic action of pGSN may be mediated by Akt and HIF1α. Triple-mutant dominant negative Akt (DN-Akt) A2780cp and A2780s cells constitutively expressing an activated Akt (A-Akt) and their respective control cells with empty vectors were cultured with and without CDDP (10 µM; 24 h) to examine the regulatory role of Akt in HIF1α and pGSN contents, as well as apoptotic response to CDDP. Upregulating Akt function in the chemosensitive cells by forced expression of A-Akt significantly increased contents of HIF1α and pGSN and attenuated CDDP-induced apoptosis (Fig. 5a). In contrast, Akt downregulation in the chemoresistant cells by DN-Akt expression resulted in decreased pGSN and HIF1α contents and facilitated CDDP-induced apoptosis (Fig. 5b).

### CDDP-induced proteasomal degradation of HIFlα regulates the pGSN content

To demonstrate if changes in HIF1α stability may influence the regulation of pGSN content, we treated A2780s cells with the proteasomal degradation inhibitor epoxomycin (10 nM; 3 h prior to CDDP treatment) or ΔHIF1α cDNA [with the degradation domain mutated (1 µg; 24 h)], and then treated with or without CDDP (10 µM; 24 h). Inhibition of HIF1α degradation by epoxomycin (Fig. 5c) or forced expression of nondegradable mutant HIF1α (Fig. 5d) in the chemosensitive cells resulted in increased pGSN content and attenuated CDDP-induced apoptosis. When HIF1α was downregulated in the resistant cells (A2780cp) in the presence of 1-methyl PA (10 µM; 3 h), a pharmacological inducer of HIF1α degradation, pGSN content was reduced and the resistant cells were sensitized to CDDP-induced apoptosis (Fig. 5e). These findings suggest that CDDP regulates pGSN content and the responsiveness of OVCA cells by modulating proteasomal HIF1α degradation.

### Chemoresistant cells-derived exosomes induce CDDP resistance to chemosensitive OVCA cells by upregulating pGSN contents

**Fig. 6:**
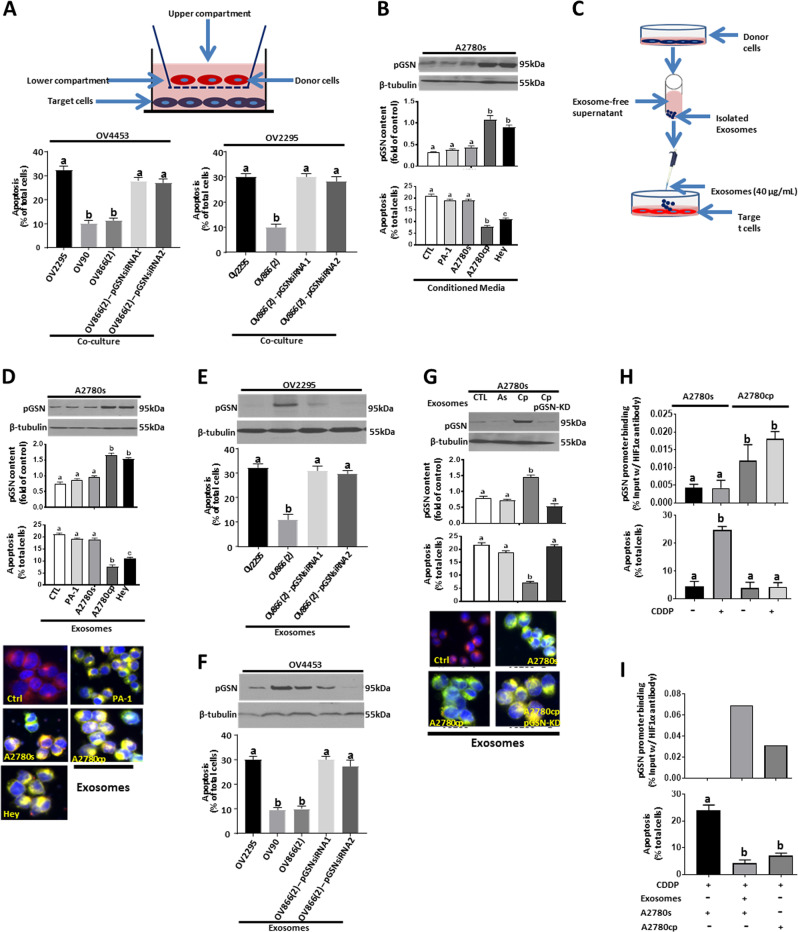
Chemoresistant cells-derived exosomes enhance HIF1α binding to pGSN promoter region and induces CDDP resistance in chemosensitive OVCA cells. **a** Chemosensitive OVCA cells (target cells; OV4453, OV2295) were co-cultured with chemoresistant OVCA cells (OV90, OV866(2)), chemosensitive OVCA cells (OV2295), and pGSN-knocked down OV866(2) cells followed by CDDP treatment (10 µm; 24 h). Chemoresistant (OV90 and OV866(2)) but not the chemosensitive OVCA cells conferred CDDP resistance to chemosensitive OVCA cells. OV866(2) cells whose pGSN was knocked down failed to protect OV4453 and OV2295 against CDDP-induced apoptosis. **b**, **c** Conditioned media and exosomes from chemoresistant OVCA cells but not the chemosensitive cells increased pGSN content and conferred CDDP resistance to chemosensitive cells. A2780s cells were treated with conditioned media (B, 3 ml; 24 h) or exosomes (**c**–**g**, 40 µg/400,000 cells; 24 h) derived from cultures of A2780s, PA-1, Hey, OV2295, OV866(2), OV90, and A2780cp cells, and then cultured with or without CDDP (10 µM; 24 h). Exosomes were tagged with pCT-CD63-GFP (1 µg; 24 h) and their uptake by recipient cells (A2780s, labeled with PKH26 red fluorescent dyes) was assessed by IF. **e**, **f** Exosomes from chemoresistant cells depleted of pGSN failed to upregulate pGSN content and facilitated CDDP-induced apoptosis compared with exosomes with pGSN. Exosomal pGSN from chemoresistant OVCA cells confer resistance in OV2295 and OV4453 cells. **g** A2780s cells were cultured with exosomes (40 µg/400,000 cells; 24 h) derived from A2780s, A2780cp, and A2780cp following pGSN knockdown (A2780cp-pGSN-KD) after which they were treated with or without CDDP (10 µM; 24 h). pGSN and β-tubulin contents (loading control) were examined by WB. **h** HIF1α-pGSN promoter binding is higher in A2780cp than A2780s cells. A2780s and A2780cp cells were cultured with or without CDDP (10 µM; 24 h) and HIF1α-pGSN promoter binding was assessed by the CHIP assay. **i** Chemoresistant cells-derived exosomes increase HIF1α-pGSN promoter binding and attenuate CDDP-induced apoptosis in chemosensitive cells. A2780s cells were cultured with A2780cp cells-derived exosomes (40 µg/400,000 cells; 24 h), and then cultured with or without CDDP (10 µM; 24 h). HIF1α-pGSN promoter binding was assessed by ChIP assay. [**a** (a; ***p < 0.001 vs b); **b** (a; ***p < 0.001 vs b and c); **d** (a; ***p < 0.001 vs b and c); **e** (a; ***p < 0.001 vs b); **f** (a; ***p < 0.001 vs b); **g** (a; ***p < 0.001 vs b); **h** (a; **p < 0.01 vs b); **i** (a; **p < 0.01 vs b)]. *N* = 3

We have examined the possibility that pGSN in chemoresistant cells-derived EXs induces CDDP resistance in chemosensitive OVCA cells. A2780s, OV2295, and OV4453 (target cells) were co-cultured with chemosensitive and chemoresistant OVCA cells (CDDP; 10 µM; Fig. 6a), conditioned media (Fig. 6b; 3 ml, 24 h), or EXs (Fig. 6c–g; 40 µg/400,000 cells, 24 h). Chemoresistant-derived conditioned media and EXs induced upregulation of pGSN content and CDDP resistance in chemosensitive OVCA cells. Immunofluorescent studies with A2780s (target cells) tagged with PKH26 (red fluorophore) and EXs tagged with GFP indicated that EXs were taken up by the cells irrespective of the chemosensitivity of the OVCA cells from which the spent media or EXs were derived, indicating that the observed phenomenon was not due to differential uptake of the EXs (Fig. 6d–g). These findings suggest that chemoresistant cells-derived EXs are capable of inducing CDDP resistance in chemosensitive OVCA cells. The EX-mediated induction of resistance in chemosensitive cells was markedly suppressed when the pGSN in the EXs isolated from chemoresistant cells was knocked down (siRNA; 50 nM, 24 h). This suggests that the attenuation of CDDP-induced apoptosis in the chemosensitive cells was a result of pGSN in the EXs from chemoresistant OVCA cells (Fig. 6g). In reciprocal studies (as control experiments), EXs from chemosensitive cells failed to alter the responsiveness of chemoresistant cells (A2780cp) to CDDP (Fig. S5A). In addition, to validate the above pGSN-effect, we knocked down pGSN (siRNA) in A2780cp cells (target cells) and co-cultured with Ex-pGSN from A2780s and A2780cp cells before CDDP treatment (10 µM; 24 h). pGSN content in pGSN-depleted-A2780cp cells was restored after co-culture with EXs derived from chemoresistant but not chemosensitive cells; the former response was associated with significant attenuation of CDDP-induced apoptosis (Fig. S5B).

In order to investigate whether HIF1α mediates the auto-induction of pGSN, we examined by chromatin immunoprecipitation (ChIP) assay the potential transcriptional regulatory role of HIF1α in pGSN expression in A2780s and A2780cp cells treated with or without CDDP (10 µM, 12 h; Fig. 6h) and Ex-pGSN (Fig. 6i). HIF1α binding to the pGSN DNA promoter was higher in the chemoresistant OVCA cells compared with their sensitive counterparts (Fig. 6h). Although CDDP treatment failed to significantly decrease HIF1α occupancy in the sensitive cells (Fig. 6h), a significant apoptosis was observed (Fig. 6h). CDDP slightly increased HIF1α occupancy in the chemoresistant cells although that was not significant (Fig. 6h); a response that was associated with attenuation of CDDP-induced apoptosis. Chemoresistant cells-derived EXs enhanced HIF1α binding to the pGSN promoter in the chemosensitive cells; CDDP treatment failed to induce apoptosis in these cells (***p < 0.001) (Fig. 6i). Taking together, our findings suggest significant evidence to support a direct regulatory role of HIF1α on pGSN auto-induction.

## Discussion

Although pGSN has been implicated in various inflammatory disorders, injuries and bacterial infections [23], whether and how it is involved in the regulation of chemosensitivity in OVCA is not known. Our present study has demonstrated that pGSN levels are higher in chemoresistant than chemosensitive OVCA cells and are reduced in the presence of CDDP in the sensitive but not the resistant cells in vitro. Gain- and lost-of-function studies support the notion that pGSN confers CDDP resistance in OVCA cells. Secreted by EX, pGSN upregulates its own expression via the α5β1 integrin-FAK-Akt-HIF1α signaling pathway and inhibits CDDP-induced apoptosis. pGSN in chemoresistant cells-derived EXs induces resistance in chemosensitive cells through exosomal uptake and the upregulation of pGSN. In addition, increased expression of pGSN in OVCA patients significantly correlates with shortened PFS.

To our knowledge, the present communication is the first report on pGSN gene expression in human cancer patients and their application for predicting clinical outcomes. Suboptimal surgical debulking is a key determinant of tumor recurrence and chemoresistance [24] hence we examined its association with pGSN expression. Our observation indicates that elevated expression of pGSN significantly correlate with poorer PFS in serous OVCA patients as well as those with suboptimal surgical debulking irrespective of chemotherapeutic composition. PFS is suggestive of the time frame for tumor recurrence [25, 26] and directly reflects the biology of the tumor hence plays a key role in chemoresistance. With the median survival observed in both cohorts, it is likely the patients under study are largely clinically platinum sensitive (PFS of at least more than 6 months). Lots of effective palliative treatments exist to manage this patient group thus, not surprising that no significant OS differences post recurrence was observed. This could in part explain why the levels of pGSN associate with a relatively minor impact on PFS. The value of the marker could help to stratify different levels of platinum sensitivities to further refine treatment recommendations and used as a prognostic marker for counseling and follow-up of these patients. In patient cohorts with more resistant tumors, pGSN is likely to have a major impact on PFS and thus could therefore serve as a potential therapeutic candidate for further mechanistic studies which could provide information to enhance patient survival. When serous patients were combined with endometroid patients, clinical significance was only observed in those treated with taxol and platinum derivatives. This observation is consistent with the heterogenous nature of OVCA cells and combinational treatments are more beneficial to OVCA patients than platinum alone. Although this observation is the first for pGSN, it supports the prognostic significance of total GSN expression in pancreatic cancers [27], gynecological cancers [9], colorectal cancers [13], and head-and-neck cancers [28]. The findings in these clinical datasets are consistent with the observation that chemoresistant OVCA cells express higher levels of pGSN compared with their sensitive counterparts. The expression of pGSN at diagnosis could therefore be used as a possible predictive marker for patient outcomes as well as a potential target for personalized therapeutics for chemoresistant OVCA.

Exosomal transport have emerged as a key mechanism of cell–cell communications [17, 18] and their contents such as Annexin A3, miR-200b, and miR-200c are significantly associated with FIGO stage [29], lymph metastasis [29], and platinum resistance [30]. Cancer-associated adipocytes and fibroblasts secrete exosomal miR-21 that attenuates CDDP-induced apoptosis and promotes CDDP resistance in OVCA cells by targeting APAF1 [20]. NAV3 is also a target for exosomal miR-21-3p in promoting CDDP resistance [31], suggesting an important role of EXs in cell–cell communication in the development of drug resistance in a plethora of cancers, including OVCA. Regardless, targeting these genes have yielded no improvement in patient survival. There is therefore the urgent need to investigate other EX-mediated mechanisms that could play an etiologic role in CDDP resistance. Here, we demonstrate a novel mechanism through which exosomal pGSN induces CDDP resistance in OVCA cells by upregulating pGSN content in chemosensitive OVCA cells. The secretion of pGSN-containing EXs by chemoresistant OVCA cells may represent an interesting opportunity to attenuate CDDP resistance in OVCA by inhibiting the transfer of exosomal pGSN. Unlike other proteins and nucleic acids [20, 31–33], pGSN promotes its own expression through exosomal transport, thus making it an unique and reliable candidate for therapeutic target. This novel phenomenon could be exploited to reverse the sensitivity of chemoresistant cells.

Aside the exosomal-mediated paracrine role of pGSN in CDDP resistance, exosomal pGSN can act in an autocrine manner to induce its own expression via α5β1 integrin signaling. In previous reports, increased α5β1 integrin expression is significantly associated with increased OVCA progression, residual disease, surgical stage, and drug resistance [34, 35]. Increased secreted phosphoprotein 1 (SPP1) also activated the integrin β1/FAK/Akt pathway, leading to cell proliferation, migration, and invasion [36]. Although pGSN interaction with α5β1 integrin through fibronectin has been shown in the in vivo assay [15], how this receptor signals in pGSN-induced chemoresistance in OVCA cells is yet to be demonstrated. Results from the current study support the hypothesis that high levels of exosomal pGSN auto-induce endogenous pGSN contents and promote CDDP resistance in OVCA cells by activating α5β1 integrin signaling. For the first time, we have shown that exosomal pGSN and rhpGSN activate with α5β1 integrin signaling cascade, induces endogenous pGSN contents and inhibits CDDP-induced apoptosis in chemosensitive cells. This autocrine signaling cascade serves as a positive feedback mechanism to increase pGSN production, thus resulting in the resistance of the cells to CDDP. This phenomenon is consistent with the observation that OVCA patients with increased α5β1 integrin expression respond poorly to treatment [35, 36] and support the notion that increased α5β1 integrin expression together with abundant supply of pGSN could fuel the proposed positive feedback mechanism of pGSN and protect cells from CDDP-induced cell death. Antagonizing the α5β1 integrin could also provide therapeutic benefits to patients since its binding to potential ligands, such as pGSN, osteopontin, and SPP1, will be abrogated. Concurrently, blocking the release of exosomal pGSN and other ligands that stimulate this signaling cascade in a similar manner could provide additional therapeutic advantages.

**Fig. 7:**
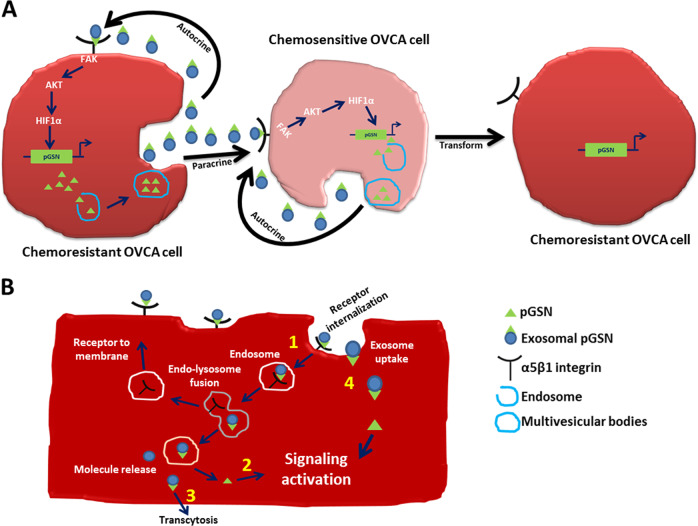
Hypothetical models illustrating the autocrine and paracrine mechanisms of Ex-pGSN in OVCA chemoresistance. **a** Chemoresistant cells (CR)-derived Ex-pGSN autoregulates its own gene expression and induces CDDP resistance in chemosensitive OVCA cells (CS) in a paracrine manner by activating the α5β1/FAK/Akt/HIF1α/pGSN signaling pathway. **b** Aside the direct activation of the α5β1/FAK/Akt/HIF1α/pGSN signaling pathway, it is also possible that exosomal pGSN-α5β1 integrin could be internalized (1) leading to the release of pGSN which further activates (2) signaling cascades resulting in chemoresistance. Upon internalization, exosomal pGSN could be released from the endosomes and secreted via transcytosis (3); a phenomenon that could propel the autocrine and paracrine mechanisms described in **a**. There is also the possibility that exosomal pGSN could be uptaken and pGSN released to activate (4) signaling cascades resulting in chemoresistance

Since α5β1 integrin is known to be mediated through a host of signaling pathways, including FAK/ERK/MAPK, RAC/NFkB, Wnt/β-catenin, and FAK/Crk/Jnk [34–37], it is possible that these pathways might be affected and thus the fate of the cells when α5β1 integrin activation is suppressed. These possibilities need to be investigated. Moreover, the possibility that the target cells for pGSN could take up exosomal pGSN via receptor-dependent and -independent endocytosis to modulate their cellular fate could not be excluded and is a subject for future investigation (Fig. 7).

The activation of α5β1 integrin cascade by exosomal pGSN also resulted in increased levels of HIF1α content. Although it well established that HIF1α overexpression is associated with OVCA tumor aggressiveness, progression and metastasis [38–40], inhibiting HIF1α as a therapeutic option has yielded no success [41]. Previous study detected by co-precipitation suggests that HIF1α binds to GSN complex although a functional link between the two proteins has not been established [38]. We have demonstrated that HIF1α knockdown decreases the content of pGSN and sensitizes chemoresistant cells to CDDP-induced death. Taking together, we hypothesized that auto-induction of pGSN is mediated via HIF1α. We investigated the binding of HIF1α to pGSN DNA promoter region in OVCA cells and for the first time HIF1α binds to the pGSN promoter and is involved in pGSN-induced pGSN expression/secretion. HIF1α occupancy was higher in the chemoresistant cells compared with their sensitive counterparts at basal levels. Although HIF1α occupancy was not significantly decreased by CDDP treatment in the sensitive cells, it was enough to induce apoptosis in these cells. Chemoresistant-derived exosomal pGSN however, increased HIF1α binding in chemosensitive cells which rendered them resistant to CDDP-induced death. HIF1α-pGSN binding motifs are currently being investigated to develop inhibitors that will disrupt their interaction, reduce pGSN production and increase responsiveness to CDDP-induced death.

In the present study, we further investigated the role of Akt in pGSN-mediated OVCA chemoresistance. We and others have previously demonstrated the important role of Akt in cell survival [5, 42, 43], protein synthesis [44, 45], and cell cycle progression and proliferation [46, 47], although whether Akt activation regulates pGSN production is not known. Upregulating Akt function in the chemosensitive cells significantly increased pGSN and HIF1α contents and attenuated CDDP-induced apoptosis, responses not observed in the resistant cells when Akt function was downregulated. In contrast, Akt activation in chemosensitive cells promotes HIF1α-mediated auto-induction of pGSN and CDDP resistance. These findings are consistent with the observation that cancer patients with increased Akt activation respond poorly to chemotherapeutic agents and have worst survival outcomes [5, 48]. Clinical trials with Akt inhibitors are currently in progress to determine its therapeutic efficiency in patients with chemoresistant OVCA [49, 50] although the role of pGSN remains uncertain. There is currently no evidence to indicate HIF1α is a substrate of Akt and sequence analysis of HIF1α indicates the absence of Akt consensus sites [51]. We therefore envisage that Akt activates HIF1α indirectly via other kinases such as CKI-Ser247 [51], GSK3β-Ser551 [51], CKII-Thr796 [51], Erk1-Ser641 [51], and PKA-Ser643 [51], possibilities worthy of future investigation.

In conclusion, we have shown for the first time that the dual functions of pGSN and that pGSN transported via EXs are a determinant of chemoresistance in OVCA cells. We have demonstrated that the autocrine action of exosomal pGSN in OVCA cells is via α5β1 integrin—HIF1α-mediated auto-induction of pGSN, a response promoted by Akt activation and resulted in CDDP resistance (Fig. 7). In addition, chemoresistant cells-derived EXs confer CDDP resistance in a paracrine manner to otherwise chemosensitive OVCA cells by increasing endogenous pGSN content. These findings support our hypothesis that exosomal pGSN promotes cancer cell survival through both autocrine and paracrine mechanisms which induces resistant phenotype in the chemosensitive cells (Fig. 7a). To our knowledge, this is the first study to demonstrate that pGSN is involved in α5β1 integrin/FAK/Akt/HIF1α/pGSN signaling cascade (Fig. 7a). Further studies are however, needed to examine other mechanisms that could be potentially implicated in pGSN-mediated chemoresistance (Fig. 7b). It is also possible that pGSN bound to α5β1 integrin could be internalized and exosomal pGSN released and secreted via transcytosis (Fig. 7b). pGSN could also be released from the EXs in the cytosol and trigger signaling cascade resulting in pGSN-mediated chemoresistance (Fig. 7b). Cancer cells may also uptake exosomal pGSN in a receptor-independent manner, release pGSN into the cytosol to activate chemoresistant signaling pathways (Fig. 7b). Although increased expression of pGSN in the ovarian tumor microenvironment correlates with poorer survival, whether it has a suppressive effect on antitumor cells is currently under investigation. Exosomal pGSN could be a clinically useful prognostic marker in the tumor microenvironment as well as offer novel insights into developing individualized treatments for chemoresistant OVCA patients.

## Materials and methods

### Ovarian cancer survival analysis

pGSN gene expression analysis was performed on primary OVCA datasets using the 200696_s_at probe specific for pGSN (Supplementary Table 1) available publicly on www.kmplot.com [22]. Patients were stratified using the following clinical parameters: histological subtypes (serous; serous and endometroid), treatments containing either platinum agents or platinum and taxol derivatives and suboptimal surgical debulking. Kaplan–Meier survival analysis was used to correlate pGSN gene expression with PFS and OS (data not shown) using optimal cutoffs in each case. Statistical parameters were calculated by log-rank test. Beeswarm plots were used to visualize gene expression in each stratified parameter.

### Kmplotter analysis

We interrogated all datasets available on kmplotter using only the 200696_s_at probe regardless of tumor stage, grade or p53 statuses. This probe specifically targets 11 sequences on the transcript variant 1 (mRNA) of GSN isoform a (pGSN; Supplementary Table 1) but not any other isoform (Supplementary Table 2). Using the optimal cutoff point, the patients were dichotomized into low-pGSN and high-pGSN groups with no specific follow-up threshold selected. The analyses were restricted to either serous or all (serous + endometroid) histological subtypes available. All databases were included in the analyses with biased array excluded. The analyses were limited to patients treated with either platinum or platinum + taxol. Unless otherwise stated (suboptimal debulking), all patients who underwent debulking surgery were included in the analyses regardless of the residual disease. Patients surviving over the specific thresholds were censored instead of being excluded.

### Reagents

CDDP, phenylmethylsulfonyl fluoride, aprotinin, dimethyl sulfoxide, sodium orthovanadate (Na_3_VO_4_), and Hoechst 33258 were supplied by Sigma (St. Louis, MO). GSN siRNA and scrambled sequence siRNA (control) were purchased from Ambion (Burlington, Canada) and Dharmacon (Colorado, USA), respectively. ΔHIF1α cDNA was purchased from Addgene (Cambridge, USA0. 1-methyl-PA, PKH67, and PKH26 Fluorescent Cell Linker Kits and epoxomycin were purchased from MilliporeSigma (Canada). siRNAs for HIF1α and FAK and their scrambled sequence siRNA (control) were supplied from Santa Cruz (Mississauga, Canada). rhpGSN was purchased from Cytoskeleton, Inc, USA. pGSN cDNA and 3.1A vector plasmids were produced in the lab of Dr Dar-Bin Shieh, National Cheng Kung University Hospital, Taiwan. pCT-CD63-GFP was purchased from System Biosciences, LLC. ATN 161 was from R&D Systems (Minnesota, USA). Sandwich pGSN ELISA kits were from Aviscera Bioscience, Inc. CA. See Supplementary Tables 4 and 5 for details on antibodies and pGSN-specific siRNA sequences, respectively. The DN-Akt and constitutively active Akt (A-Akt) adenoviruses were generous gifts from Dr Kenneth Walsh (Boston University School of Medicine, Boston, MA) and have been routinely used in our laboratory [42, 43]. The DN-Akt is a triple mutant with a dead-kinase [with an alanine at the Thr308 and Ser473 (required in phosphorylation for Akt activation [52, 53] and at Lys179 (within the kinase domain and required for phosphate transfer [54]]. The A-Akt is a myristoylated Akt2. The availability of HA-tagged DN-Akt and HA-tagged A-Akt virus allow for easy confirmation of their expression by western blot.

### Cell lines and cell culture

Chemosensitive and chemoresistant OVCA cell lines of HGS and endometroid histologic subtypes and with various p53 status were tested in the present studies: HGS [OV2295 (p53-mutant; sensitive), OV4453 (p53-mutant, sensitive), OV90 (p53-mutant, resistant), OV866(2) (p53-mutant, resistant), Hey (p53-wt, resistant)], and Endometroid [A2780s (p53-wt, sensitive), PA-1 (p53-wt; sensitive), A2780cp (p53-mutant, resistant), SKOV-3 (p53-null, resistant)]. The OVCA cell lines were cultured in Dulbecco’s modified Eagle medium/F12 and RPMI 1640 as previously reported [8, 43]. All experiments were carried out in serum-free media. See supplementary Table 3 for details on cells.

### RNA interference

Cells were transfected (50–200 nM, 24 h) with siRNAs for total GSN [(Ambion and Dharmacon; HIF1α and FAK (Santa Cruz)] or their scrambled control, using lipofectamine 2000, and were subsequently treated with CDDP (10 µM; 24 h) and harvested for analysis, as previously described [8, 43, 55]. siRNA against total GSN targets the C terminal region which is shared by both cGSN and pGSN. However, siRNA against pGSN specifically targets and degrades pGSN mRNA without affecting that of cGSN. Two different siRNAs (Supplementary Table 5) were used for each target to exclude off-target effects. Successful knockdown was confirmed by western blotting (WB) [9] (see Supplementary Table 4 for details on antibodies).

### Transient transfection

Cells were transfected with pGSN and ΔHIF1α pcDNA3.1-derived vectors (empty vector as controls), using lipofectamine 2000, and were then cultured with CDDP (10 µM; 24 h) and harvested for further analysis [8, 43, 55]. Successful overexpression was confirmed by WB (see Supplementary Table 4 on antibodies).

### ChIP assay

ChIP assays were performed on human OVCA cells, as previously described [56]. The antibodies and primers used for verifying the occupancy of HIF1α on the pGSN promoter are shown in the Supplementary Tables 4 and 6, respectively. Sequence −3000 base pairs upstream of TSS was analyzed for potential promoter and based on the score of the promoter strength, primers were designed to determine HIF1a binding (Supplementary Table 6).

### Extracellular vesicle isolation and characterization

Serum-free conditioned media from cultured cells were used for EV isolation and characterization, as described [57]. Total EV concentration was determined by BCA the Protein Assay Kit (Thermo Fisher Scientific). When fresh EXs (40 µg/400,000 cells) were not required, they were suspended in PBS and stored at −80 °C for subsequent analysis.

### Nanoparticle tracking analysis

EVs in PBS were analyzed, using the ZetaView PMX110 Multiple Parameter Particle Tracking Analyzer (Particle Metrix, Meerbusch, Germany) in size mode using ZetaView software version 8.02.28, as previously described [57, 58]. EVs were captured at 11 camera positions at 21 °C and pellet size and concentration evaluated.

### Transmission electron microscopy (TEM)

OVCA cell was pelleted (4000 g; 20 min) and processed, as previously described [59]. Resin sections were stained with uranyl acetate and lead citrate solutions and examined with a Jeol JEM 1230 transmission electron microscope (Japan).

### Immunoelectron microscopy (iEM)

Cell pellets (4000 g; 20 min) were processed as previously described [59]. The grids were washed three times in PBST, immunostained with anti-pGSN antibody (Supplementary Table 4), rinsed in distilled water, stained with uranyl acetate and lead citrate, and photographed with a Jeol JEM 1230 transmission electron microscope (Japan).

### Exosome labeling, uptake and fluorescent microscopy

Donor cells were transfected with pCT-CD63-GFP to label the EXs (1 µg; 24 h), while recipient cells were labeled with PKH26 red fluorescent dyes (Sigma-Aldrich, MO), as previously described [57, 58]. Nuclei were counterstained with DAPI, cells were mounted onto coverslips and EX uptake examined, as previously described [57, 58].

### Protein extraction and western blot analysis

WB was carried out as previously described [8, 9, 43]. Membranes containing transferred proteins were incubated with primary antibodies (1:1000) in 5% (wt/vol) Blotto and subsequently with the appropriate horseradish peroxidase-conjugated secondary antibody (1:2000) in 5% (wt/vol) Blotto (Supplementary Table 4 for details on antibodies). Peroxidase activity was visualized on a film with the Enhanced Chemiluminescent Kit (Amersham Biosciences) and signal intensity densitometrically determined (Image J software).

### ELISA

pGSN contents in cell-free conditioned media and lysates from OVCA cells were assayed by the sandwich ELISA (Aviscera Bioscience, Inc. CA), according to the manufacturer’s instructions.

### Assessment of apoptosis

CDDP-induced apoptosis was assessed morphologically [8, 55], using Hoechst 33258 nuclear stain. Using a random selection of fields, a minimum of 400 cells with typical apoptotic nuclear morphology (nuclear condensation, shrinkage, and fragmentation) were counted in each group and expressed as the percentage of total cells. “Blinded” counting approach was used to prevent experimental bias.

### Statistical analyses

Results are expressed as the mean ± SD of at least three independent experiments. Statistical analysis was carried out by one- or two-way ANOVA and differences between multiple experimental groups were determined by Bonferroni post hoc test, using the PRISM software (Version 7.0; GraphPad, San Diego, CA). Statistical significance was inferred at *p* < 0.05. Statistical parameters for Kaplan–Meier survival analysis were calculated by log-rank.

## Supplementary information

Supplementary Tables

Supplementary Figures
