# Supplementary material for: The exosome-mediated autocrine and paracrine actions of plasma gelsolin in ovarian cancer chemoresistance
Source: Oncogene. 2019 Nov 7;39(7):1600–16. doi: 10.1038/s41388-019-1087-9 (PMC7018662; doi:10.1038/s41388-019-1087-9)
Supplement: Supplementary file 1 — Supplementary Tables [file 41388_2019_1087_MOESM1_ESM.docx]

| Probe Set Name | Probe X | Probe Y | Probe Interrogation Position | Probe Sequence | Target Strandedness |
| --- | --- | --- | --- | --- | --- |
| 200696_s_at | 310 | 575 | 2122 | TGCTTCTGGACACCTGGGACCAGGT | Antisense |
| 200696_s_at | 702 | 477 | 2137 | GGGACCAGGTCTTTGTCTGGGTTGG | Antisense |
| 200696_s_at | 451 | 343 | 2190 | GAAGCCTTGACTTCTGCTAAGCGGT | Antisense |
| 200696_s_at | 613 | 621 | 2207 | TAAGCGGTACATCGAGACGGACCCA | Antisense |
| 200696_s_at | 375 | 569 | 2266 | TGAAGCAAGGCTTTGAGCCTCCCTC | Antisense |
| 200696_s_at | 509 | 297 | 2299 | GCTGGTTCCTTGGCTGGGATGATGA | Antisense |
| 200696_s_at | 528 | 551 | 2321 | TGATTACTGGTCTGTGGACCCCTTG | Antisense |
| 200696_s_at | 334 | 607 | 2398 | TCACCGGTCAGTGCCTTTTGGAACT | Antisense |
| 200696_s_at | 611 | 229 | 2421 | CTGTCCTTCCCTCAAAGAGGCCTTA | Antisense |
| 200696_s_at | 59 | 63 | 2436 | AGAGGCCTTAGAGCGAGCAGAGCAG | Antisense |
| 200696_s_at | 204 | 381 | 2455 | GAGCAGCTCTGCTATGAGTGTGTGT | Antisense |

**Supplementary Table 1: Target sequences for 200696_s_at probe and GSN transcript variant 1; mRNA (Isoform a; pGSN) sequence.**

**Homo sapiens gelsolin (GSN), transcript variant 1, mRNA (Isoform a); probe target sequences are highlighted in yellow.**

1 ccaccatggc tccgcaccgc cccgcgcccg cgctgctttg cgcgctgtcc ctggcgctgt

61 gcgcgctgtc gctgcccgtc cgcgcggcca ctgcgtcgcg gggggcgtcc caggcggggg

121 cgccccaggg gcgggtgccc gaggcgcggc ccaacagcat ggtggtggaa caccccgagt

181 tcctcaaggc agggaaggag cctggcctgc agatctggcg tgtggagaag ttcgatctgg

241 tgcccgtgcc caccaacctt tatggagact tcttcacggg cgacgcctac gtcatcctga

301 agacagtgca gctgaggaac ggaaatctgc agtatgacct ccactactgg ctgggcaatg

361 agtgcagcca ggatgagagc ggggcggccg ccatctttac cgtgcagctg gatgactacc

421 tgaacggccg ggccgtgcag caccgtgagg tccagggctt cgagtcggcc accttcctag

481 gctacttcaa gtctggcctg aagtacaaga aaggaggtgt ggcatcagga ttcaagcacg

541 tggtacccaa cgaggtggtg gtgcagagac tcttccaggt caaagggcgg cgtgtggtcc

601 gtgccaccga ggtacctgtg tcctgggaga gcttcaacaa tggcgactgc ttcatcctgg

661 acctgggcaa caacatccac cagtggtgtg gttccaacag caatcggtat gaaagactga

721 aggccacaca ggtgtccaag ggcatccggg acaacgagcg gagtggccgg gcccgagtgc

781 acgtgtctga ggagggcact gagcccgagg cgatgctcca ggtgctgggc cccaagccgg

841 ctctgcctgc aggtaccgag gacaccgcca aggaggatgc ggccaaccgc aagctggcca

901 agctctacaa ggtctccaat ggtgcaggga ccatgtccgt ctccctcgtg gctgatgaga

961 accccttcgc ccagggggcc ctgaagtcag aggactgctt catcctggac cacggcaaag

1021 atgggaaaat ctttgtctgg aaaggcaagc aggcaaacac ggaggagagg aaggctgccc

1081 tcaaaacagc ctctgacttc atcaccaaga tggactaccc caagcagact caggtctcgg

1141 tccttcctga gggcggtgag accccactgt tcaagcagtt cttcaagaac tggcgggacc

1201 cagaccagac agatggcctg ggcttgtcct acctttccag ccatatcgcc aacgtggagc

1261 gggtgccctt cgacgccgcc accctgcaca cctccactgc catggccgcc cagcacggca

1321 tggatgacga tggcacaggc cagaaacaga tctggagaat cgaaggttcc aacaaggtgc

1381 ccgtggaccc tgccacatat ggacagttct atggaggcga cagctacatc attctgtaca

1441 actaccgcca tggtggccgc caggggcaga taatctataa ctggcagggt gcccagtcta

1501 cccaggatga ggtcgctgca tctgccatcc tgactgctca gctggatgag gagctgggag

1561 gtacccctgt ccagagccgt gtggtccaag gcaaggagcc cgcccacctc atgagcctgt

1621 ttggtgggaa gcccatgatc atctacaagg gcggcacctc ccgcgagggc gggcagacag

1681 cccctgccag cacccgcctc ttccaggtcc gcgccaacag cgctggagcc acccgggctg

1741 ttgaggtatt gcctaaggct ggtgcactga actccaacga tgcctttgtt ctgaaaaccc

1801 cctcagccgc ctacctgtgg gtgggtacag gagccagcga ggcagagaag acgggggccc

1861 aggagctgct cagggtgctg cgggcccaac ctgtgcaggt ggcagaaggc agcgagccag

1921 atggcttctg ggaggccctg ggcgggaagg ctgcctaccg cacatcccca cggctgaagg

1981 acaagaagat ggatgcccat cctcctcgcc tctttgcctg ctccaacaag attggacgtt

2041 ttgtgatcga agaggttcct ggtgagctca tgcaggaaga cctggcaacg gatgacgtca

2101 tgcttctgga cacctgggac caggtctttg tctgggttgg aaaggattct caagaagaag

2161 aaaagacaga agccttgact tctgctaagc ggtacatcga gacggaccca gccaatcggg

2221 atcggcggac gcccatcacc gtggtgaagc aaggctttga gcctccctcc tttgtgggct

2281 ggttccttgg ctgggatgat gattactggt ctgtggaccc cttggacagg gccatggctg

2341 agctggctgc ctgaggaggg gcagggccca cccatgtcac cggtcagtgc cttttggaac

2401 tgtccttccc tcaaagaggc cttagagcga gcagagcagc tctgctatga gtgtgtgtgt

2461 gtgtgtgtgt tgtttctttt tttttttttt acagtatcca aaaatagccc tgcaaaaatt

2521 cagagtcctt gcaaaattgt ctaaaatgtc agtgtttggg aaattaaatc caataaaaac

2581 attttgaagt gtgaaaaaaa aaaaaaaaaa aaaaaaaaaa aaaaaaaaaa aaaaaaaaaa

2641 aaaaaaaaaa aaaaaaaaaa aaa

**Supplementary Table 2: Target sequences for 214040_s_at probe and GSN transcript variant 4; mRNA (Isoform f) sequence.**

| Probe Set Name | Probe X | Probe Y | Probe Interrogation Position | Probe Sequence | Target Strandedness |
| --- | --- | --- | --- | --- | --- |
| 214040_s_at | 136 | 103 | 262 | ACAGCATGGTGGTGGAACACCCCGA | Antisense |
| 214040_s_at | 627 | 313 | 265 | GCATGGTGGTGGAACACCCCGAGTT | Antisense |
| 214040_s_at | 615 | 535 | 311 | TGGCCTGCAGATCTGGCGTGTGGAG | Antisense |
| 214040_s_at | 407 | 319 | 317 | GCAGATCTGGCGTGTGGAGAAGTTC | Antisense |
| 214040_s_at | 55 | 549 | 329 | TGTGGAGAAGTTCGATCTGGTGCCC | Antisense |
| 214040_s_at | 360 | 235 | 353 | CGTGCCCACCAACCTTTATGGAGAC | Antisense |
| 214040_s_at | 156 | 175 | 359 | CACCAACCTTTATGGAGACTTCTTC | Antisense |
| 214040_s_at | 104 | 141 | 408 | AAGACAGTGCAGCTGAGGAACGGAA | Antisense |
| 214040_s_at | 153 | 105 | 411 | ACAGTGCAGCTGAGGAACGGAAATC | Antisense |
| 214040_s_at | 433 | 23 | 433 | ATCTGCAGTATGACCTCCACTACTG | Antisense |
| 214040_s_at | 377 | 573 | 436 | TGCAGTATGACCTCCACTACTGGCT | Antisense |

**Homo sapiens gelsolin (GSN), transcript variant 4, mRNA (Isoform f); probe target sequences are highlighted in yellow.**

1 ggaaccagct gagcgcagct ggacccagca gccgctgtct ccagtgccgc agcagcaggt

61 agtgctcata gctctctttg tccagtgctt cggccttggt cccagcgcct tcccacggag

121 cagcactctt caccctgcac agccttgtta ggagaagggg atgaatgaat acaggactta

181 cgcgtctgct gtggcccagc tggcttccag atggtgacat gagccaccca cagccggagc

241 tgttcctctt tcccaaagct cagcccaaca gcatggtggt ggaacacccc gagttcctca

301 aggcagggaa ggagcctggc ctgcagatct ggcgtgtgga gaagttcgat ctggtgcccg

361 tgcccaccaa cctttatgga gacttcttca cgggcgacgc ctacgtcatc ctgaagacag

421 tgcagctgag gaacggaaat ctgcagtatg acctccacta ctggctgggc aatgagtgca

481 gccaggatga gagcggggcg gccgccatct ttaccgtgca gctggatgac tacctgaacg

541 gccgggccgt gcagcaccgt gaggtccagg gcttcgagtc ggccaccttc ctaggctact

601 tcaagtctgg cctgaagtac aagaaaggag gtgtggcatc aggattcaag cacgtggtac

661 ccaacgaggt ggtggtgcag agactcttcc aggtcaaagg gcggcgtgtg gtccgtgcca

721 ccgaggtacc tgtgtcctgg gagagcttca acaatggcga ctgcttcatc ctggacctgg

781 gcaacaacat ccaccagtgg tgtggttcca acagcaatcg gtatgaaaga ctgaaggcca

841 cacaggtgtc caagggcatc cgggacaacg agcggagtgg ccgggcccga gtgcacgtgt

901 ctgaggaggg cactgagccc gaggcgatgc tccaggtgct gggccccaag ccggctctgc

961 ctgcaggtac cgaggacacc gccaaggagg atgcggccaa ccgcaagctg gccaagctct

1021 acaaggtctc caatggtgca gggaccatgt ccgtctccct cgtggctgat gagaacccct

1081 tcgcccaggg ggccctgaag tcagaggact gcttcatcct ggaccacggc aaagatggga

1141 aaatctttgt ctggaaaggc aagcaggcaa acacggagga gaggaaggct gccctcaaaa

1201 cagcctctga cttcatcacc aagatggact accccaagca gactcaggtc tcggtccttc

1261 ctgagggcgg tgagacccca ctgttcaagc agttcttcaa gaactggcgg gacccagacc

1321 agacagatgg cctgggcttg tcctaccttt ccagccatat cgccaacgtg gagcgggtgc

1381 ccttcgacgc cgccaccctg cacacctcca ctgccatggc cgcccagcac ggcatggatg

1441 acgatggcac aggccagaaa cagatctgga gaatcgaagg ttccaacaag gtgcccgtgg

1501 accctgccac atatggacag ttctatggag gcgacagcta catcattctg tacaactacc

1561 gccatggtgg ccgccagggg cagataatct ataactggca gggtgcccag tctacccagg

1621 atgaggtcgc tgcatctgcc atcctgactg ctcagctgga tgaggagctg ggaggtaccc

1681 ctgtccagag ccgtgtggtc caaggcaagg agcccgccca cctcatgagc ctgtttggtg

1741 ggaagcccat gatcatctac aagggcggca cctcccgcga gggcgggcag acagcccctg

1801 ccagcacccg cctcttccag gtccgcgcca acagcgctgg agccacccgg gctgttgagg

1861 tattgcctaa ggctggtgca ctgaactcca acgatgcctt tgttctgaaa accccctcag

1921 ccgcctacct gtgggtgggt acaggagcca gcgaggcaga gaagacgggg gcccaggagc

1981 tgctcagggt gctgcgggcc caacctgtgc aggtggcaga aggcagcgag ccagatggct

2041 tctgggaggc cctgggcggg aaggctgcct accgcacatc cccacggctg aaggacaaga

2101 agatggatgc ccatcctcct cgcctctttg cctgctccaa caagattgga cgttttgtga

2161 tcgaagaggt tcctggtgag ctcatgcagg aagacctggc aacggatgac gtcatgcttc

2221 tggacacctg ggaccaggtc tttgtctggg ttggaaagga ttctcaagaa gaagaaaaga

2281 cagaagcctt gacttctgct aagcggtaca tcgagacgga cccagccaat cgggatcggc

2341 ggacgcccat caccgtggtg aagcaaggct ttgagcctcc ctcctttgtg ggctggttcc

2401 ttggctggga tgatgattac tggtctgtgg accccttgga cagggccatg gctgagctgg

2461 ctgcctgagg aggggcaggg cccacccatg tcaccggtca gtgccttttg gaactgtcct

2521 tccctcaaag aggccttaga gcgagcagag cagctctgct atgagtgtgt gtgtgtgtgt

2581 gtgttgtttc tttttttttt ttttacagta tccaaaaata gccctgcaaa aattcagagt

2641 ccttgcaaaa ttgtctaaaa tgtcagtgtt tgggaaatta aatccaataa aaacattttg

2701 aagtgtg

**Supplementary Table 3. Information on OVCA cell lines**

| **Cell line** | **Tumor origin** | **TP53 status** | **Other** | **Chemosensitivity** |
| --- | --- | --- | --- | --- |
| A2780s | Ovarian endometroid adenocarcinoma | Wild type | PTEN/ARID1A | Sensitive |
| A2780cp | Ovarian endometroid adenocarcinoma | Mutant  V127F, R260S | PTEN/ARID1A | Resistant |
| SKOV3 | Ovarian endometroid adenocarcinoma | Null | PI3KCIA/ARID1A | Resistant |
| PA-1 | Ovarian endometroid adenocarcinoma | Wild type | None Detected | Sensitive |
| Hey | Ovarian serous cyst  adenocarcinoma | Wild type | KRAS | Resistant |
| OV2295 | High grade serous ovarian cancer | Mutant  IIe195Thr | Non Detected | Sensitive |
| OV4453 | High grade serous ovarian cancer | Mutant  Splice | None Detected | Sensitive |
| OV90 | High grade serous ovarian cancer | Mutant  Ser215Arg | None Detected | Resistant |
| OV866(2) | High grade serous ovarian cancer | Mutant  Arg249Trp | None Detected | Resistant |

**Information on OVCA cell lines**: The characterization of these cell lines have been verified in previous literature (Anglesio et al.,2013; Leroy et al., 2014; Provencher et al., 2000; Fleury et al., 2015; Letourneau et al., 2012).

| **Primary Antibodies** | | | | | | **Secondary Antibodies** | | | | | |
| --- | --- | --- | --- | --- | --- | --- | --- | --- | --- | --- | --- |
| **Application** | **Target** | **Antibody** | **Company** | **Catalog #** | **Dilution** | **Antibody** | **Conjugate** | **Company** | **Catalog #** | **Dilution** | **Note** |
| WB | pGSN | Anti-pGSN Goat polyclonal | Antibodies online(Atlanta,USA) | ABIN1019662 | 1:1000 | Dnk polyclonal to Goat IgG | HRP | Abcam (Toronto, Canada) | Ab97110 | 1:2000 |  |
| WB | cGSN | Anti-GSN mouse monoclonal | MilliporeSigma (Oakville, Canada) | G4896 | 1:1000 | Goat Anti-mouse IgG (H+L) | HRP | Bio-Rad (Mississauga, Canada) | 170-6516 | 1:2000 |  |
| WB | HIF1α | Anti- HIF1α mouse monoclonal | MilliporeSigma (Oakville, Canada) | H6536 | 1:1000 | Goat Anti-mouse IgG (H+L) | HRP | Bio-Rad (Mississauga, Canada) | 170-6516 | 1:2000 |  |
| WB | CD63 | Anti-CD63 mouse monoclonal | Abcam (Toronto, Canada) | Ab193349 | 1:1000 | Goat Anti-mouse IgG (H+L) | HRP | Bio-Rad (Mississauga, Canada) | 170-6516 | 1:2000 |  |
| WB | FAK | Anti-FAK mouse monoclonal | MilliporeSigma (Oakville, Canada) | 396500 | 1:1000 | Goat Anti-mouse IgG (H+L) | HRP | Bio-Rad (Mississauga, Canada) | 170-6516 | 1:2000 |  |
| WB | HA | Anti-HA mouse monoclonal | Santa-Cruz (Mississauga, Canada) | Sc-805 | 1:1000 | Goat Anti-mouse IgG (H+L) | HRP | Bio-Rad (Mississauga, Canada) | 170-6516 | 1:2000 |  |
| WB | β-tubulin | Anti- β-tubulin mouse monoclonal | MilliporeSigma (Oakville, Canada) | SAB4200715 | 1:1000 | Goat Anti-mouse IgG (H+L) | HRP | Bio-Rad (Mississauga, Canada) | 170-6516 | 1:2000 |  |
| ChIP | HIF1α | Anti- HIF1α mouse monoclonal | MilliporeSigma (Oakville, Canada) | H6536 | 5 µg | N/A |  |  |  |  | Used in Fig. 6H. |
| ChIP | HIF1α | Anti- HIF1α mouse monoclonal | Novus Biologicals (Oakville, Canada) | NB100-105 | 5 µg | N/A |  |  |  |  | Used in Fig. 6I. |
| iEM | pGSN | Anti-pGSN mouse monoclonal | ABGENT (San Diego, US) | AM1936a | 1:100 | Goat-anti-mouse-IgG | Colloidal gold | Jackson (PA, USA) | 115-215-068 | 1:50 |  |

Supplementary Table 4: Antibodies used in the present studies

**Supplementary Table 5: Customized siRNA oligonucleotide duplexes**

| **Product** | **Target** | **Species** | **Company** | **Catalog #** | **Target sequence** | **Anti-sense sequence** | **Position on mRNA** |
| --- | --- | --- | --- | --- | --- | --- | --- |
| siRNA 1 | pGSN | Human | IDT (Iowa, USA) | N/A | GCGACCCGAGGCCGCGGCU | AGCCGCGGCCUCGGGUCGC | 12 |
| siRNA 2 | pGSN | Human | IDT (Iowa, USA) | N/A | UGCCCGAGGCGCGGCCCAA | UUGGGCCGCGCCUCGGGCA | 192 |

**Supplementary Table 6:  primer sequences for amplifying the gelsolin promoter.**

HIF1a_pGSN FP: GGCCCATGTATATGTCCTGA

HIF1a_pGSN RP: AGTTTGGGCTTCAGCAACAG
