# Supplementary material for: The exosome-mediated autocrine and paracrine actions of plasma gelsolin in ovarian cancer chemoresistance
Source: Oncogene. 2019 Nov 7;39(7):1600–16. doi: 10.1038/s41388-019-1087-9 (PMC7018662; doi:10.1038/s41388-019-1087-9)
Supplement: Supplementary file 2 — Supplementary Figures [file 41388_2019_1087_MOESM2_ESM.pptx]

## Slide 1
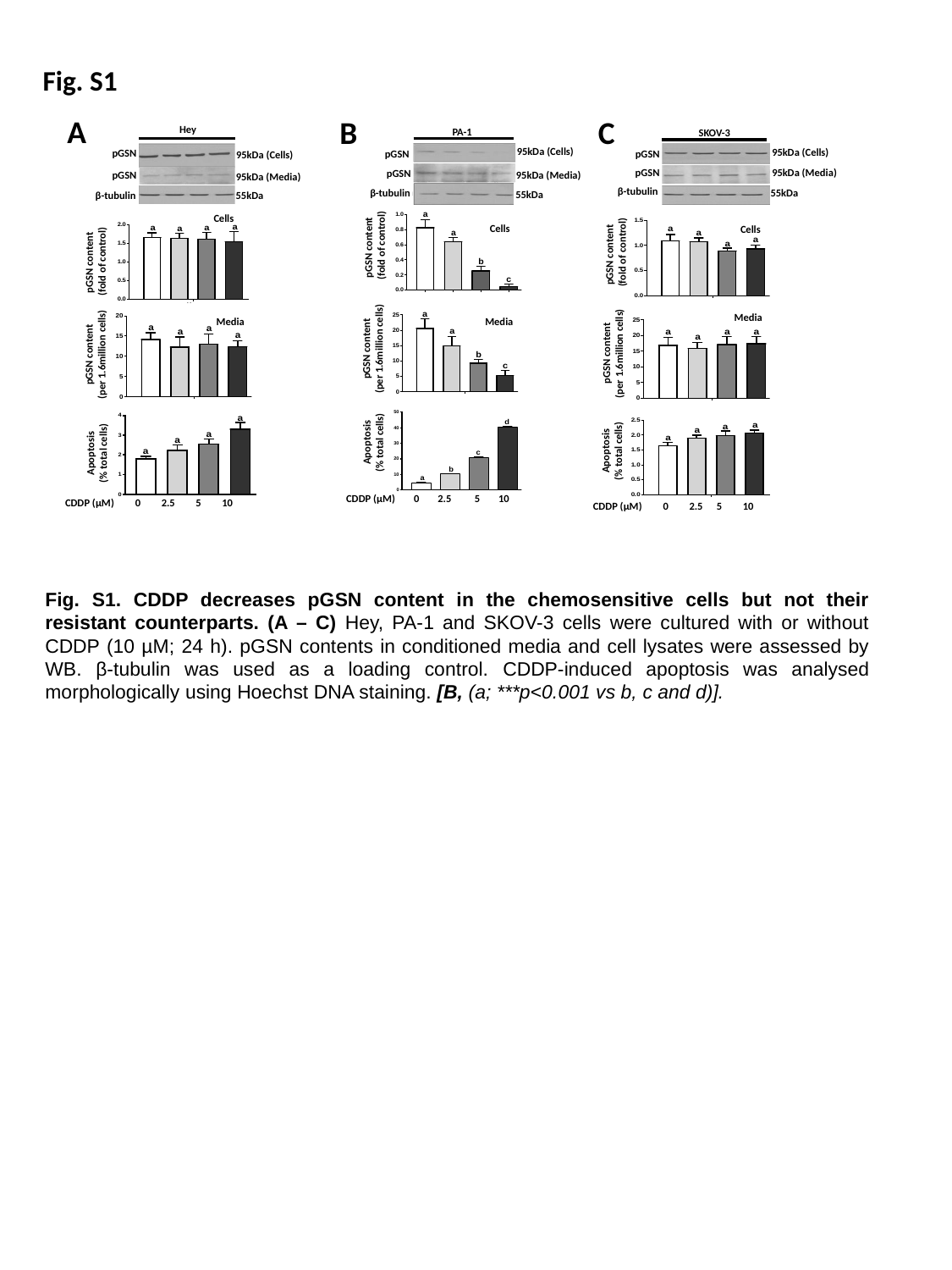

Fig. S1
A
B
C
Hey
pGSN
95kDa (Cells)
pGSN
95kDa (Media)
β-tubulin
pGSN content
(fold of control)
Apoptosis
(% total cells)
pGSN content
(per 1.6million cells)
CDDP (µM) 0 2.5 5 10
Cells
Media
PA-1
95kDa (Cells)
pGSN
pGSN
95kDa (Media)
β-tubulin
pGSN content
 (fold of control)
pGSN content
(per 1.6million cells)
Apoptosis
(% total cells)
CDDP (µM) 0 2.5 5 10
Cells
Media
SKOV-3
95kDa (Cells)
pGSN
95kDa (Media)
pGSN
β-tubulin
pGSN content
 (fold of control)
Apoptosis
(% total cells)
pGSN content
(per 1.6million cells)
CDDP (µM) 0 2.5 5 10
Cells
Media
55kDa
55kDa
55kDa
Fig. S1. CDDP decreases pGSN content in the chemosensitive cells but not their resistant counterparts. (A – C) Hey, PA-1 and SKOV-3 cells were cultured with or without CDDP (10 µM; 24 h). pGSN contents in conditioned media and cell lysates were assessed by WB. β-tubulin was used as a loading control. CDDP-induced apoptosis was analysed morphologically using Hoechst DNA staining. [B, (a; ***p<0.001 vs b, c and d)].

## Slide 2
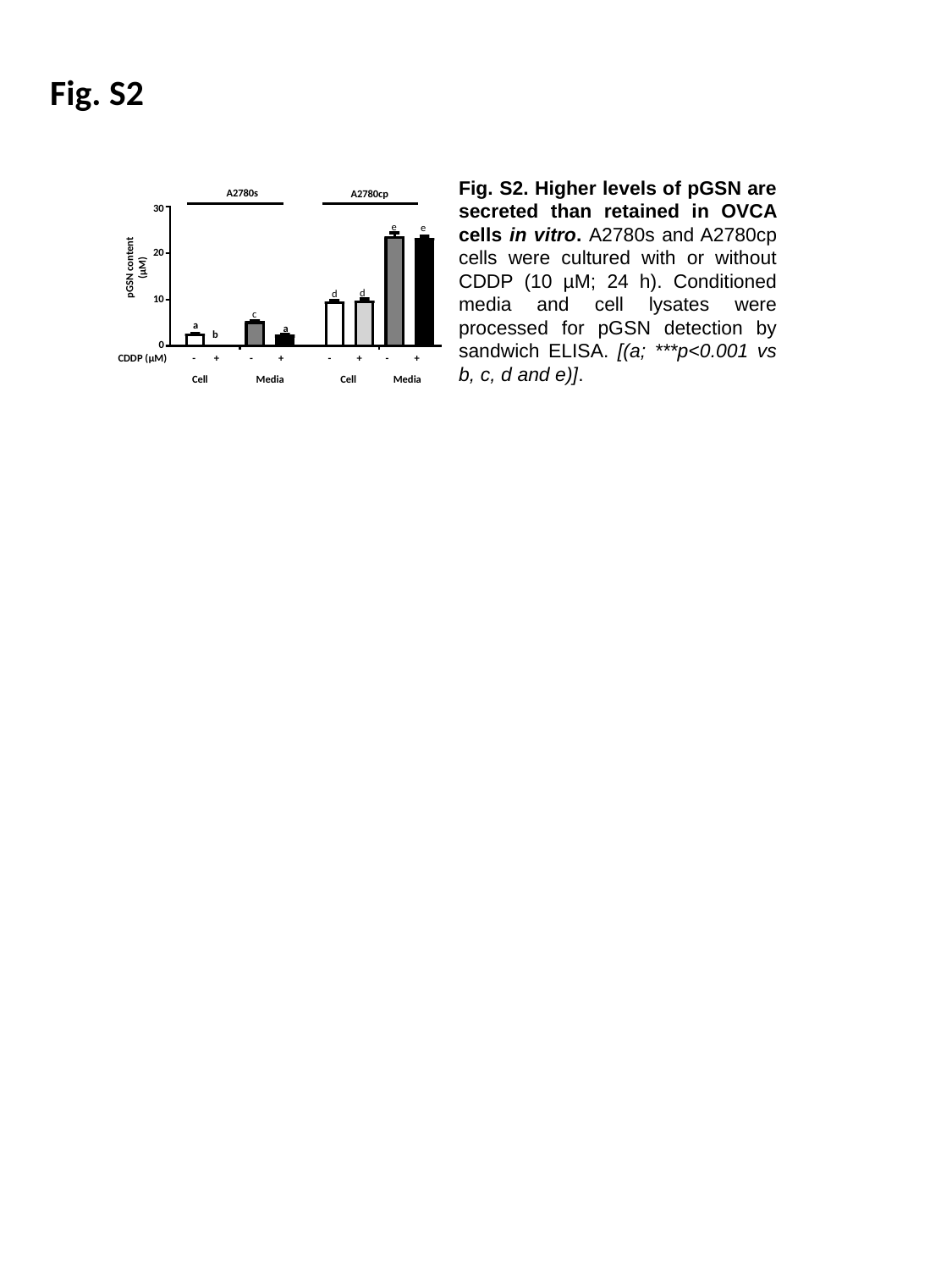

Fig. S2
Fig. S2. Higher levels of pGSN are secreted than retained in OVCA cells in vitro. A2780s and A2780cp cells were cultured with or without CDDP (10 µM; 24 h). Conditioned media and cell lysates were processed for pGSN detection by sandwich ELISA. [(a; ***p<0.001 vs b, c, d and e)].
A2780s
A2780cp
30
e
e
20
pGSN content
(μM)
d
d
10
c
a
a
b
0
Cell
Media
Cell
Media
CDDP (μM)
- + - + - + - +

## Slide 3
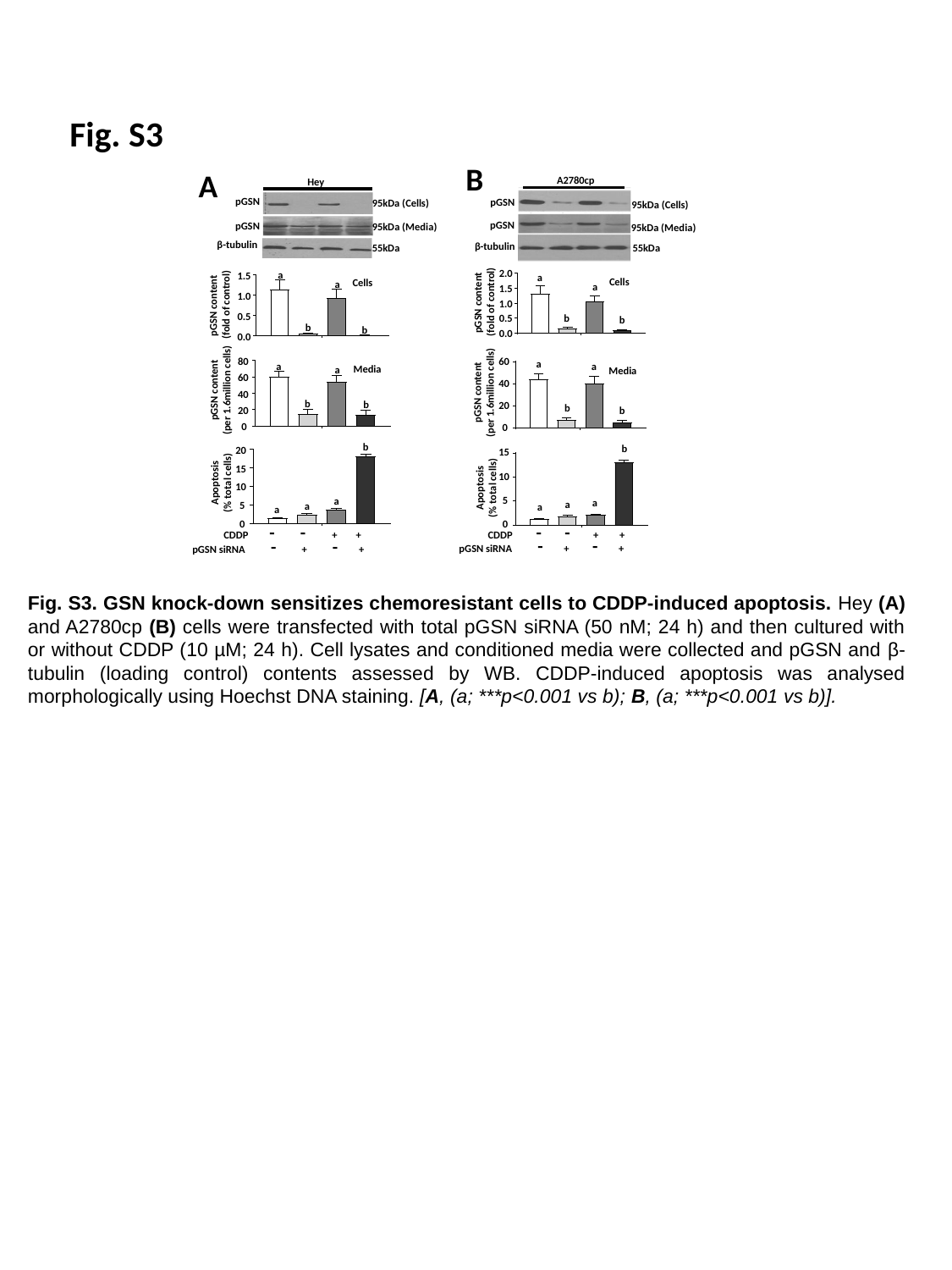

# Fig. S3
B
A2780cp
pGSN
95kDa (Cells)
pGSN
95kDa (Media)
β-tubulin
55kDa
2.0
1.5
1.0
0.5
0.0
60
40
20
0
15
10
5
0
a
a
b
b
a
a
b
b
b
a
a
a
Cells
pGSN content (fold of control)
Media
pGSN content
(per 1.6million cells)
Apoptosis
(% total cells)
CDDP - - + +
pGSN siRNA - + - +
A
Hey
pGSN
95kDa (Cells)
pGSN
95kDa (Media)
β-tubulin
55kDa
a
1.5
Cells
a
pGSN content (fold of control)
1.0
0.5
b
b
0.0
80
a
a
60
40
b
b
20
0
Media
pGSN content
(per 1.6million cells)
b
20
15
10
a
5
a
a
0
Apoptosis
(% total cells)
CDDP - - + +
pGSN siRNA - + - +
Fig. S3. GSN knock-down sensitizes chemoresistant cells to CDDP-induced apoptosis. Hey (A) and A2780cp (B) cells were transfected with total pGSN siRNA (50 nM; 24 h) and then cultured with or without CDDP (10 µM; 24 h). Cell lysates and conditioned media were collected and pGSN and β-tubulin (loading control) contents assessed by WB. CDDP-induced apoptosis was analysed morphologically using Hoechst DNA staining. [A, (a; ***p<0.001 vs b); B, (a; ***p<0.001 vs b)].

## Slide 4
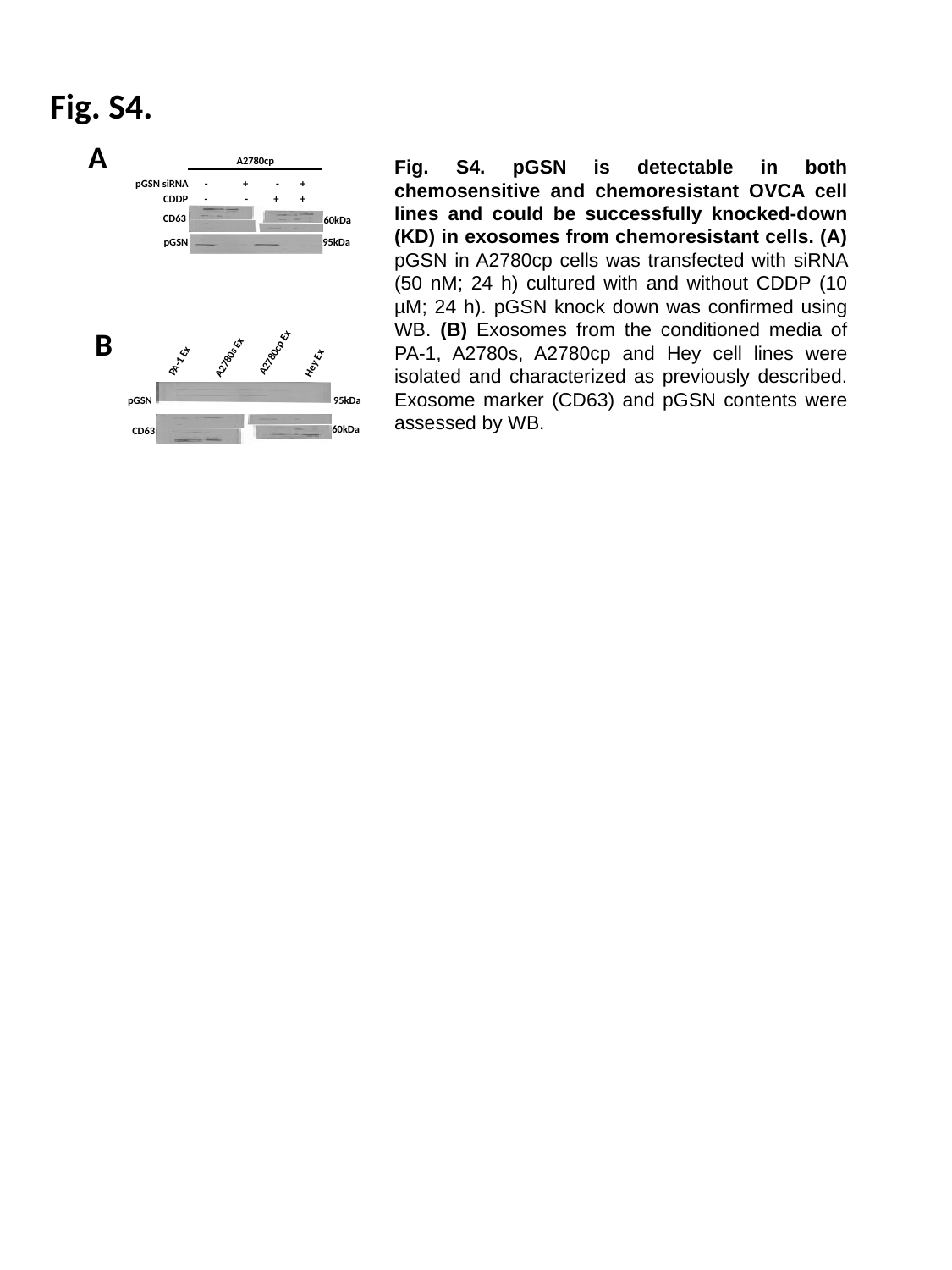

# Fig. S4.
A
A2780cp
 pGSN siRNA - + - +
CDDP - - + +
CD63
pGSN
A2780cp Ex
A2780s Ex
PA-1 Ex
Hey Ex
pGSN
CD63
B
Fig. S4. pGSN is detectable in both chemosensitive and chemoresistant OVCA cell lines and could be successfully knocked-down (KD) in exosomes from chemoresistant cells. (A) pGSN in A2780cp cells was transfected with siRNA (50 nM; 24 h) cultured with and without CDDP (10 µM; 24 h). pGSN knock down was confirmed using WB. (B) Exosomes from the conditioned media of PA-1, A2780s, A2780cp and Hey cell lines were isolated and characterized as previously described. Exosome marker (CD63) and pGSN contents were assessed by WB.
60kDa
95kDa
95kDa
60kDa

## Slide 5
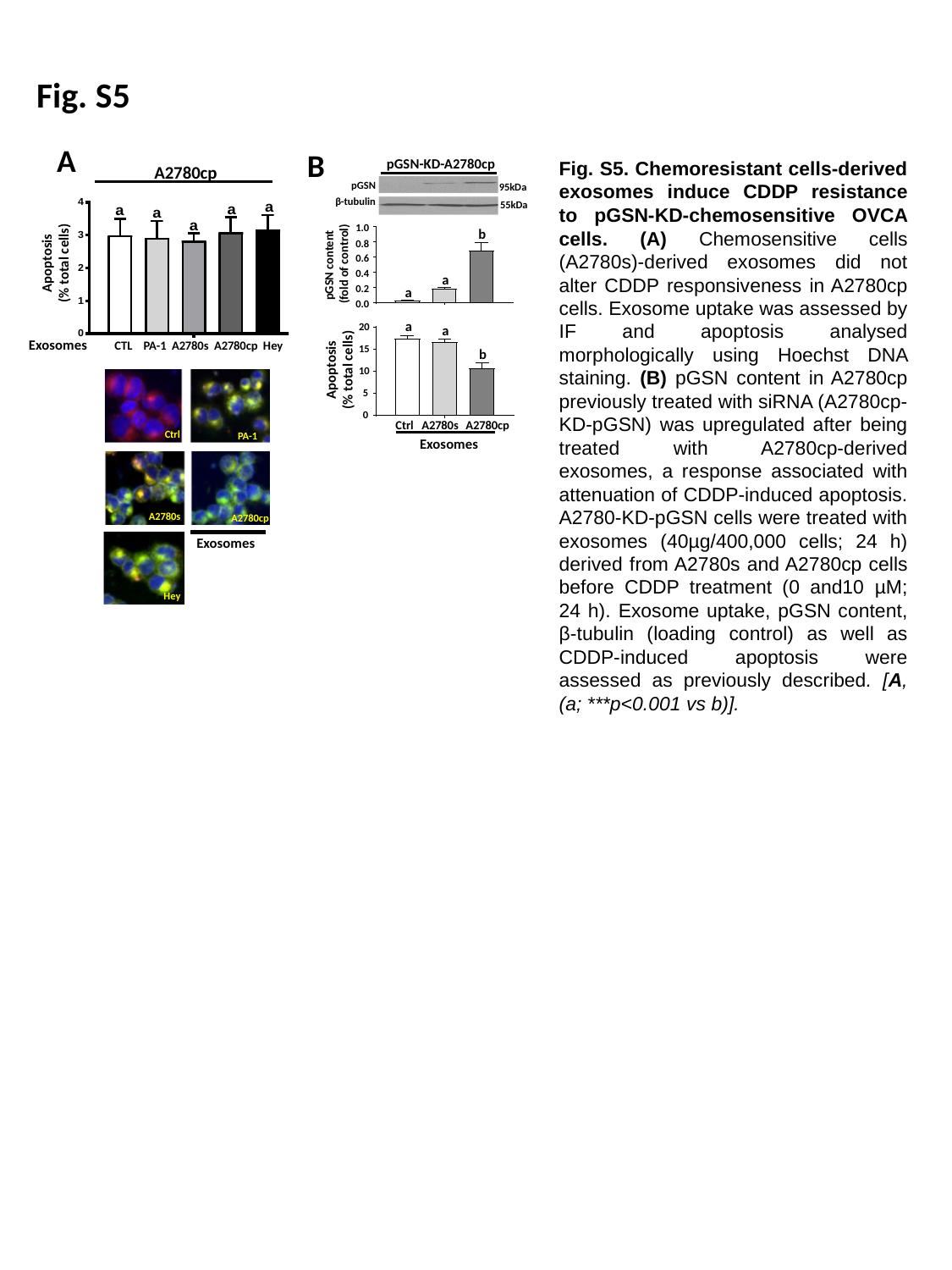

Fig. S5
A
A2780cp
Apoptosis
(% total cells)
Ctrl
PA-1
A2780s
A2780cp
Exosomes
Hey
Exosomes
CTL PA-1 A2780s A2780cp Hey
B
pGSN-KD-A2780cp
pGSN
β-tubulin
1.0
0.8
0.6
0.4
0.2
0.0
b
pGSN content (fold of control)
a
a
a
20
15
10
5
0
a
b
Apoptosis
(% total cells)
Ctrl
A2780s
A2780cp
Exosomes
Fig. S5. Chemoresistant cells-derived exosomes induce CDDP resistance to pGSN-KD-chemosensitive OVCA cells. (A) Chemosensitive cells (A2780s)-derived exosomes did not alter CDDP responsiveness in A2780cp cells. Exosome uptake was assessed by IF and apoptosis analysed morphologically using Hoechst DNA staining. (B) pGSN content in A2780cp previously treated with siRNA (A2780cp-KD-pGSN) was upregulated after being treated with A2780cp-derived exosomes, a response associated with attenuation of CDDP-induced apoptosis. A2780-KD-pGSN cells were treated with exosomes (40µg/400,000 cells; 24 h) derived from A2780s and A2780cp cells before CDDP treatment (0 and10 µM; 24 h). Exosome uptake, pGSN content, β-tubulin (loading control) as well as CDDP-induced apoptosis were assessed as previously described. [A, (a; ***p<0.001 vs b)].
95kDa
55kDa
